# Preoperative CT-derived skeletal muscle phenotypes, postoperative complication burden, and long-term survival after liver transplantation: a 90-day landmark mediation analysis

**DOI:** 10.3389/fnut.2026.1978429

**Published:** 2026-09-28

**Authors:** Rui Yu, Bin Sun, Junjie Du, Lizhi Lv

**Affiliations:** 1 Fuzong Clinical Medical College of Fujian Medical University, Fuzhou, Fujian, China; 2 Department of Hepatobiliary and Pancreatic Surgery, 900th Hospital of PLA Joint Logistic Support Force, Fuzhou, Fujian, China

**Keywords:** body composition, clinical nutrition, Comprehensive Complication Index, liver transplantation, mediation analysis, postoperative complications, skeletal muscle density, skeletal muscle index

## Abstract

**Background:**

CT-derived skeletal muscle index (SMI) and skeletal muscle density (SMD) may complement nutritional and functional assessment before liver transplantation, but pathways linking these body-composition phenotypes to long-term outcomes remain unclear. We evaluated whether cumulative 90-day postoperative complication burden was compatible with an intermediate pathway between lower preoperative SMI or SMD and long-term mortality.

**Methods:**

This single-center retrospective cohort used a postoperative day-90 landmark, restricting the primary analysis to recipients alive and under follow-up at day 90. SMI and SMD were analyzed separately, with the 90-day Comprehensive Complication Index (CCI) as the mediator and all-cause mortality during a prespecified 5-year post-landmark window as the outcome. Linear mediator and Cox outcome models adjusted for hepatocellular carcinoma, Eastern Cooperative Oncology Group performance status, and MELD 3.0. Percentile 95% confidence intervals (CIs) were obtained from 1,000 nonparametric bootstrap resamples.

**Results:**

Of 245 recipients screened, 205 entered the landmark cohort; 56 (27.3%) died during the prespecified 5-year post-landmark analysis window. Median analyzed follow-up was 3.33 years. For a 1-standard deviation decrease, the approximate natural indirect-effect hazard ratios (HRs) through CCI were 1.155 (95% bootstrap CI, 1.033–1.326) for SMI and 1.190 (1.062–1.382) for SMD. Natural direct-effect CIs included 1 for both exposures, whereas the total-effect CI excluded 1 for SMI but not SMD. Indirect-effect estimates were similar after replacing ECOG with age and delaying outcome follow-up to day 120, but were attenuated after CCI dichotomization.

**Conclusion:**

Lower preoperative SMI and SMD showed model-based approximate indirect effects through cumulative postoperative complication burden. These CT-derived muscle phenotypes may complement, but should not replace, comprehensive nutritional and functional assessment. The observational design and incompletely verifiable identification assumptions preclude strict causal or interventional interpretation.

## 1 Introduction

Liver transplantation (LT) is an established treatment for end-stage liver disease and selected hepatic malignancies meeting transplant criteria. Despite advances in surgical and perioperative care, long-term overall survival varies among recipients and is associated with graft-related factors as well as recipient physiological reserve and frailty (1, 2). Chronic inflammation, metabolic disturbances, inadequate nutritional intake, and accelerated muscle protein breakdown in end-stage liver disease can alter body composition and reduce tolerance to perioperative stress (3, 4). Objective assessment of skeletal muscle quantity and composition may therefore complement broader clinical, nutritional, and functional evaluation during pretransplant risk stratification.

Skeletal muscle abnormalities are common in end-stage liver disease and can be quantified opportunistically using routinely acquired computed tomography (CT). At the third lumbar vertebral level, skeletal muscle index (SMI) provides a height-normalized measure of muscle area, whereas skeletal muscle density (SMD) represents the mean CT attenuation of the segmented muscle area and is commonly used as an imaging marker of muscle composition; lower values are associated with myosteatosis (5). Because abdominal CT is routinely obtained during transplant evaluation, these measures can provide opportunistic body-composition information without additional imaging. Neither measure alone establishes malnutrition or sarcopenia. The Global Leadership Initiative on Malnutrition (GLIM) framework requires nutritional-risk screening followed by at least one phenotypic and one etiologic criterion for a diagnosis of malnutrition, whereas the revised European Working Group on Sarcopenia in Older People (EWGSOP2) defines low muscle strength as probable sarcopenia, low muscle quantity or quality as confirmation, and poor physical performance as an indicator of severe sarcopenia (6, 7). Because the complete GLIM assessment and measures of muscle strength and physical performance were unavailable, SMI and SMD were treated as CT-derived muscle phenotypes rather than diagnostic labels. Lower SMI and SMD may characterize body-composition vulnerability in the setting of nutritional and metabolic disturbances and reduced physiological resilience (3–5) and have been associated with infection, surgical complications, prolonged hospitalization, and poorer survival after LT (5, 8). Nevertheless, previous studies have focused mainly on prognosis, and the pathways linking preoperative skeletal muscle phenotypes to long-term survival remain insufficiently examined.

Postoperative complication burden may represent one such pathway. The Clavien-Dindo classification grades individual complications according to treatment intensity but does not capture the cumulative burden of multiple postoperative events. Derived from Clavien-Dindo grades, the Comprehensive Complication Index (CCI) integrates all complications during a defined period into a weighted continuous score ranging from 0 to 100 (9). Higher CCI has been associated with graft loss at 90 days and 1 year and with poorer long-term survival after LT (10, 11). However, skeletal muscle phenotypes, postoperative complication burden, and survival have generally been studied separately. Evaluating statistical mediation through CCI may help clarify whether the observed associations are compatible with an intermediate clinical pathway. Under relevant identification assumptions, counterfactual mediation methods estimate natural direct and indirect effects; in observational data, however, such decompositions remain model- and assumption-dependent (12).

Using a retrospective cohort with a postoperative day-90 landmark, we evaluated whether the associations of lower preoperative CT-derived SMI and SMD with all-cause mortality during a prespecified 5-year post-landmark analysis window were compatible with an indirect pathway through cumulative CCI accrued from completion of liver transplantation to immediately before the day-90 landmark. We hypothesized that lower SMI and SMD would be associated with greater postoperative complication burden and that model-based analyses would show indirect effects through CCI.

## 2 Materials and methods

### 2.1 Study design, setting, and landmark cohort

This single-center retrospective cohort screened consecutive patients undergoing first liver transplantation at the 900th Hospital of PLA Joint Logistic Support Force, Fuzhou, China, from January 2016 through December 2025. Patients were excluded when preoperative CT data were inadequate for skeletal muscle assessment or when key clinical data required for the planned analyses were unavailable.

A postoperative day-90 landmark was prespecified to separate mediator ascertainment from outcome follow-up and preserve their temporal ordering (13, 14). After these exclusions, the remaining recipients constituted the baseline eligible cohort. The primary landmark cohort was restricted to recipients who were alive and under follow-up on postoperative day 90. Recipients who died before postoperative day 90 or whose recorded follow-up ended before day 90 were therefore excluded from the landmark analysis.

### 2.2 Preoperative CT-derived skeletal muscle assessment

The most recent routine abdominal CT examination obtained within 3 months before liver transplantation was used for skeletal muscle assessment. A single axial image at the mid-level of the third lumbar vertebra (L3) was selected. Skeletal muscle was automatically segmented using uAI software (United Imaging Intelligence, Shanghai, China), after which trained image readers reviewed each segmentation and performed manual correction when necessary. Within the segmented muscle regions, pixels with CT attenuation values from −29 to +150 Hounsfield units (HU) were included in skeletal muscle quantification (5). Additional image-selection, segmentation, and quality-control procedures are described in the Supplementary Methods (Section 1.1).

Skeletal muscle index (SMI) was calculated as the total cross-sectional skeletal muscle area at L3 divided by height squared and was expressed in cm^2^/m^2^. Skeletal muscle density (SMD) was defined as the mean CT attenuation of the segmented skeletal muscle area and was expressed in HU. SMI and SMD were analyzed separately and were not simultaneously included as exposures in the same primary model. Each exposure was standardized as a z score within the primary day-90 landmark cohort; the prespecified exposure contrast was a 1-standard deviation decrease.

### 2.3 Postoperative complication burden and data verification

Cumulative postoperative complication burden was measured using the Comprehensive Complication Index (CCI) from completion of liver transplantation to immediately before the postoperative day-90 landmark. The CCI integrates all qualifying Clavien-Dindo grade I or higher complications during a defined period into a continuous score ranging from 0 to 100 (9, 15).

Distinct and independently identifiable complications were entered separately. When the management or severity of the same complication escalated, only its highest final Clavien-Dindo grade was retained. Detailed complication definitions, event-recording rules, and CCI calculation procedures are provided in the Supplementary Methods (Section 1.2).

Two physicians, blinded to preoperative SMI and SMD measurements and post-landmark survival outcome, independently reviewed the postoperative complication records for each recipient during the mediator-ascertainment window. Disagreements were resolved through adjudication by a third physician. The final adjudicated CCI records were then cross-checked against the primary analysis database on a patient-by-patient basis. This cross-check assessed agreement between the final adjudicated CCI record and the value used for analysis and was not a measure of pre-adjudication inter-rater reliability.

### 2.4 Outcome definition and follow-up

The outcome was all-cause mortality during a prespecified 5-year analysis window beginning at the postoperative day-90 landmark. Follow-up time was measured from the day-90 landmark until death, the last confirmed follow-up, or 5 years after the landmark, whichever occurred first. Observations extending beyond 5 years were administratively censored at the 5-year endpoint.

Dates of death and last confirmed follow-up were ascertained from hospital inpatient and outpatient records and longitudinal transplant follow-up records, supplemented by telephone follow-up when necessary. The database lock for outcome ascertainment was March 31, 2026. Recipients without a documented death were right-censored at their last confirmed follow-up if this occurred before the 5-year endpoint; recipients remaining under observation beyond 5 years were administratively censored at 5 years.

### 2.5 Conceptual causal framework and covariate specification

An expanded conceptual directed acyclic graph (DAG) was developed on the basis of published evidence, clinical knowledge, and temporal ordering (16). In separate exposure-specific frameworks, preoperative SMI or SMD was defined as the exposure, cumulative CCI accrued before the day-90 landmark as the mediator, and all-cause mortality during the prespecified 5-year post-landmark analysis window as the outcome.

The expanded DAG additionally represented age, sex, a conceptual domain of pre-existing non-hepatic comorbidity and metabolic burden, and donor graft characteristics as potentially relevant factors. Hepatocellular carcinoma (HCC) status, Eastern Cooperative Oncology Group (ECOG) performance status, and Model for End-Stage Liver Disease (MELD) 3.0 score were prespecified as measured covariates and included in both the mediator and outcome models. MELD 3.0 was calculated according to its published definition (17).

The primary measured covariates were specified considering clinical relevance, data availability, the number of post-landmark deaths, and model parsimony. The additional factors depicted in the expanded DAG were not jointly included in the primary models, and the three measured covariates were not assumed to account for all potential confounding pathways. Nutritional status, systemic inflammation, frailty components not captured by ECOG performance status, donor quality, and other perioperative determinants were not comprehensively measured or modeled and remained potential sources of residual or unmeasured confounding. The assumptions required to identify natural direct and indirect effects are described in the Supplementary Methods (Section 2).

### 2.6 Statistical analysis

Continuous variables were summarized as means with standard deviations or medians with interquartile ranges, as appropriate to their distributions. Categorical variables were summarized as counts and percentages. There were no missing data for variables included in the primary analyses in the final day-90 landmark cohort; therefore, no participants were excluded because of missing primary-analysis variables and no imputation was required. No *a priori* sample-size calculation was performed; the available study size was determined by the consecutive eligible recipients during the study period and subsequent eligibility for the day-90 landmark cohort.

To describe potential differences associated with day-90 landmark eligibility, perioperative and donor characteristics were compared between recipients excluded before the landmark after formation of the baseline cohort and those included in the landmark cohort. Wilcoxon rank-sum tests were used for cold ischemia time, estimated blood loss, anhepatic phase, portal vein clamping time, and inferior vena cava clamping time, whereas Welch’s t test was used for donor age. These comparisons are reported in Supplementary Table S1.

For descriptive survival presentation, SMI and SMD were separately dichotomized at their cohort medians, and Kaplan–Meier curves were compared using log-rank tests. The groups were labelled as below the cohort median and at or above the cohort median; these pooled cohort medians were used solely for descriptive visualization and were not diagnostic cutoffs. Continuous standardized SMI and SMD were retained for the primary mediation models.

Separate regression-based mediation analyses within a counterfactual framework were performed for SMI and SMD. Ordinary least squares regression was used for the continuous cumulative CCI mediator, and Cox proportional hazards regression was used for time to all-cause mortality during the prespecified 5-year post-landmark analysis window. For each exposure, the mediator model regressed cumulative CCI on the standardized exposure, HCC status, ECOG performance status, and MELD 3.0 score. The Cox outcome model included the standardized exposure, cumulative CCI, and the same three measured covariates. The primary Cox outcome models did not include an exposure-by-CCI interaction; this interaction was evaluated in a sensitivity analysis.

Natural direct effects (NDEs), natural indirect effects (NIEs), and total effects (TEs) were estimated for the prespecified contrast of a 1-standard deviation decrease in SMI or SMD (12, 18, 19). Effects were reported as hazard ratios (HRs). Because post-landmark mortality was not rare, the HR-scale natural-effect estimates were interpreted cautiously as approximate mediation effects rather than exact decompositions of risk ratios.

All mediation-effect estimates in the primary, sensitivity, and subgroup analyses were obtained using 1,000 nonparametric bootstrap resamples, with percentile-based 95% confidence intervals derived from the bootstrap distributions. Exploratory mediational E-values were calculated for the NIEs by treating the HR-scale estimates as approximate risk ratios and were interpreted only as sensitivity measures for potential unmeasured mediator-outcome confounding (20, 21).

Robustness and sensitivity analyses included replacing ECOG performance status with age in both the mediator and outcome models; adding an exposure-by-CCI interaction to the outcome model; dichotomizing CCI at its sample 75th percentile; repeating the mediation analyses in the HCC subgroup; exploratorily evaluating effect modification by sex; and delaying the start of outcome follow-up to postoperative day 120 while retaining CCI accrued from transplantation to immediately before day 90. Because the dataset did not contain a mutually exclusive classification of underlying liver-disease etiology, an additional exploratory analysis evaluated whether the SMI–CCI and SMD–CCI associations differed by HCC status (HCC vs. non-HCC), an available broad disease/indication category. The corresponding exposure-by-HCC interaction term was added to each adjusted linear mediator model, and nested models were compared using F tests. In an additional *post hoc* sensitivity analysis, low SMI was classified using published sex-specific FLEXIT/Carey thresholds (men <50 cm^2^/m^2^ and women <39 cm^2^/m^2^). This was treated as an external-threshold low-SMI classification rather than a diagnosis of sarcopenia because muscle-strength and physical-performance measures were unavailable. No analogous external-threshold analysis was performed for SMD because a universally accepted sex-specific cutoff for this liver-transplant setting was unavailable. Detailed specifications are provided in the Supplementary Methods (Sections 3.2 and 3.6).

The proportional hazards assumption was assessed using scaled Schoenfeld residuals, variable-specific tests, and global tests (22). The OLS mediator models were evaluated using residual diagnostics, including assessments of heteroscedasticity, residual normality, and influential observations; detailed procedures are provided in the Supplementary Methods (Section 3.3). Statistical analyses were performed in R version 4.5.2, primarily using the survival, regmedint, and boot packages. All hypothesis tests were two-sided, and *p* < 0.05 was considered statistically significant.

ChatGPT (GPT-5.6 Sol; OpenAI, San Francisco, CA, USA) was used to assist with drafting and checking R code during manuscript preparation. All AI-assisted code was reviewed by the authors, and all analyses were executed and validated by the authors, who independently assessed the resulting outputs and interpretations.

### 2.7 Ethical approval and organ source

This retrospective study was reviewed and approved by the Ethics Committee of the 900th Hospital of PLA Joint Logistic Support Force (approval no. 2026-183; approval date: 3 August 2026). The study was conducted in accordance with the Declaration of Helsinki (23). The Ethics Committee waived the requirement for written informed consent for participation in this retrospective study because de-identified clinical data were analyzed.

All liver transplantations included in this study were performed in the Department of Hepatobiliary and Pancreatic Surgery at the 900th Hospital of PLA Joint Logistic Support Force, Fuzhou, China. Organ procurement was coordinated by the Organ Procurement Organization (OPO) of the 900th Hospital of PLA Joint Logistic Support Force in accordance with applicable Chinese regulations and institutional procedures. For deceased donors without a documented lifetime objection to organ donation, the donation decision was made jointly in writing by the donor’s spouse, adult children, and parents, as applicable, in accordance with Chinese regulations. Before organ procurement, the donation and procurement process was reviewed by the institutional human organ transplantation ethics committee in accordance with applicable regulations. The transplantation and organ procurement procedures complied with the principles of the Declaration of Istanbul (24). All liver grafts were obtained through legal and voluntary deceased donation and were allocated through the China Organ Transplant Response System (COTRS) (25). The study involved no commercial transplantation activity and included no organs obtained from executed prisoners or other nonvoluntary or unethical sources.

## 3 Results

### 3.1 Patient flow and baseline characteristics

Of 245 recipients initially screened, 12 were excluded because preoperative CT data were inadequate for skeletal muscle assessment or key clinical data required for the planned analyses were unavailable, leaving a baseline eligible cohort of 233. A further 28 recipients were excluded from the day-90 landmark cohort: 25 died before postoperative day 90 and 3 had recorded follow-up ending before day 90. The final landmark cohort comprised 205 recipients (Figure 1).

**Figure 1:**
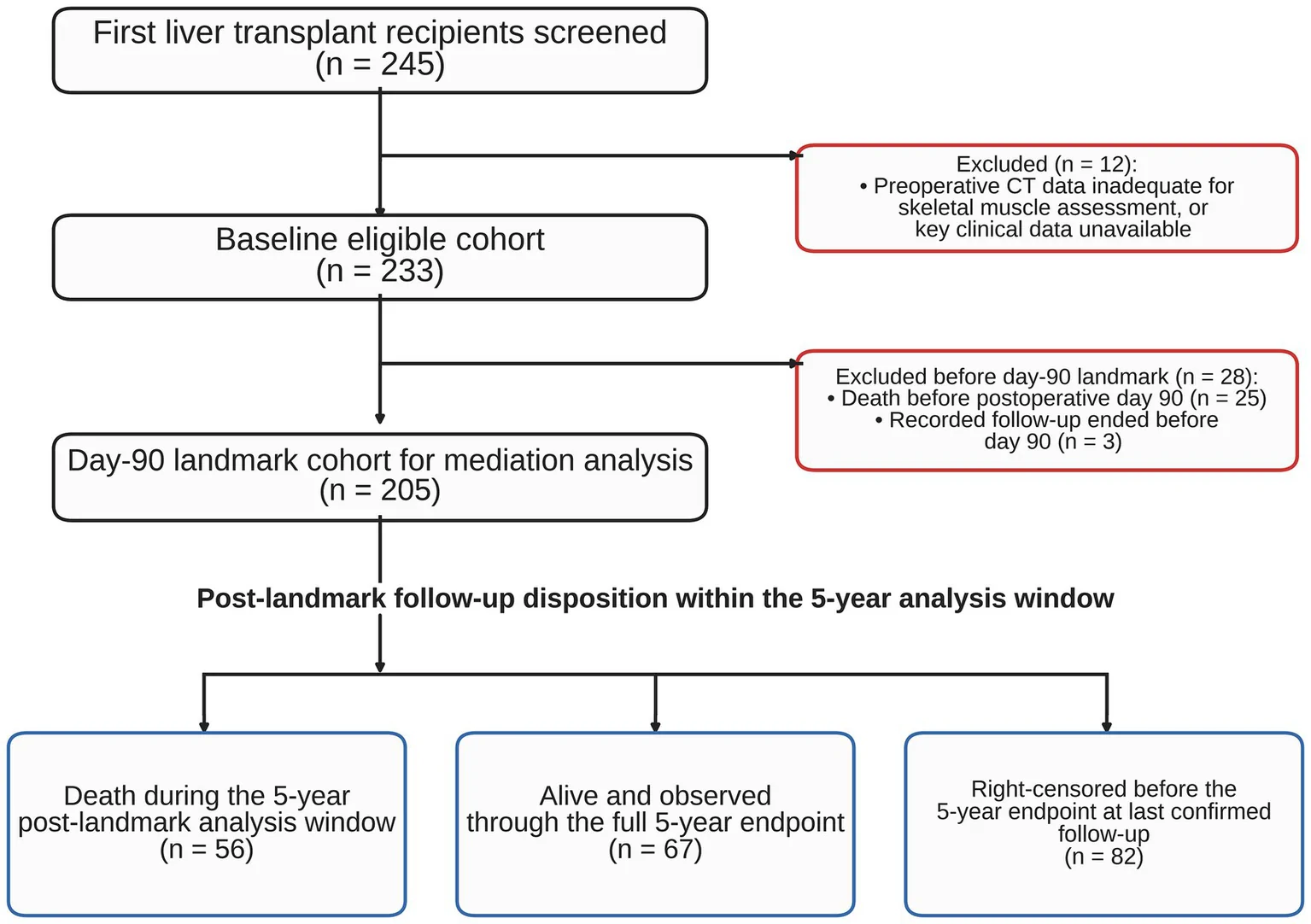
Study flowchart, construction of the postoperative day-90 landmark cohort, and post-landmark follow-up disposition. A total of 245 first liver transplant recipients were screened. After exclusion of 12 recipients because preoperative CT data were inadequate for skeletal muscle assessment or key clinical data required for the planned analyses were unavailable, 233 comprised the baseline eligible cohort. A further 28 recipients were excluded before the landmark: 25 died before postoperative day 90 and 3 had recorded follow-up ending before day 90. The final landmark cohort comprised 205 recipients. During the prespecified 5-year post-landmark analysis window, 56 recipients died, 67 were observed alive through the full 5-year endpoint, and 82 were right-censored before the 5-year endpoint at their last confirmed follow-up. All 205 recipients were retained in the time-to-event analyses.

The mean age was 49.35 ± 10.11 years, and 28 recipients (13.7%) were female. Mean body mass index, SMI, and SMD were 24.06 ± 3.58 kg/m^2^, 49.90 ± 8.23 cm^2^/m^2^, and 39.10 ± 6.34 HU, respectively. Among male and female recipients, mean SMI was 50.12 ± 8.31 and 48.55 ± 7.71 cm^2^/m^2^, respectively, and mean SMD was 39.14 ± 6.43 and 38.86 ± 5.83 HU, respectively; complete sex-specific distributions are reported in Supplementary Table S6. Overall, 113 recipients (55.1%) had HCC, 179 (87.3%) had cirrhosis, and the median MELD 3.0 score was 15 (interquartile range, 10–23; Table 1). Compared with the landmark cohort, recipients excluded before the landmark had higher estimated intraoperative blood loss (median, 2000.0 vs. 1500.0 mL; *p* = 0.016), whereas the other evaluated perioperative and donor characteristics showed no statistical evidence of between-group differences (Supplementary Table S1). The expanded conceptual DAG is shown in Figure 2.

**Table 1:** Baseline characteristics of the postoperative day-90 landmark cohort.

Characteristic	Analysis cohort (*n* = 205)
Age, years	49.35 (10.11)
Female sex	28 (13.7)
Body mass index, kg/m^2^	24.06 (3.58)
Skeletal muscle index, cm^2^/m^2^	49.90 (8.23)
Skeletal muscle density, HU	39.10 (6.34)
Hepatocellular carcinoma	113 (55.1)
Cirrhosis	179 (87.3)
ECOG performance status	
0	116 (56.6)
1	64 (31.2)
2	9 (4.4)
3	7 (3.4)
4	9 (4.4)
MELD 3.0 score	15 [10, 23]

Data are presented as mean (SD), median [IQR], or *n* (%). MELD 3.0 is presented as median [IQR]; all other continuous variables are presented as mean (SD). ECOG, Eastern Cooperative Oncology Group; HU, Hounsfield units; IQR, interquartile range; MELD, Model for End-Stage Liver Disease.

**Figure 2:**
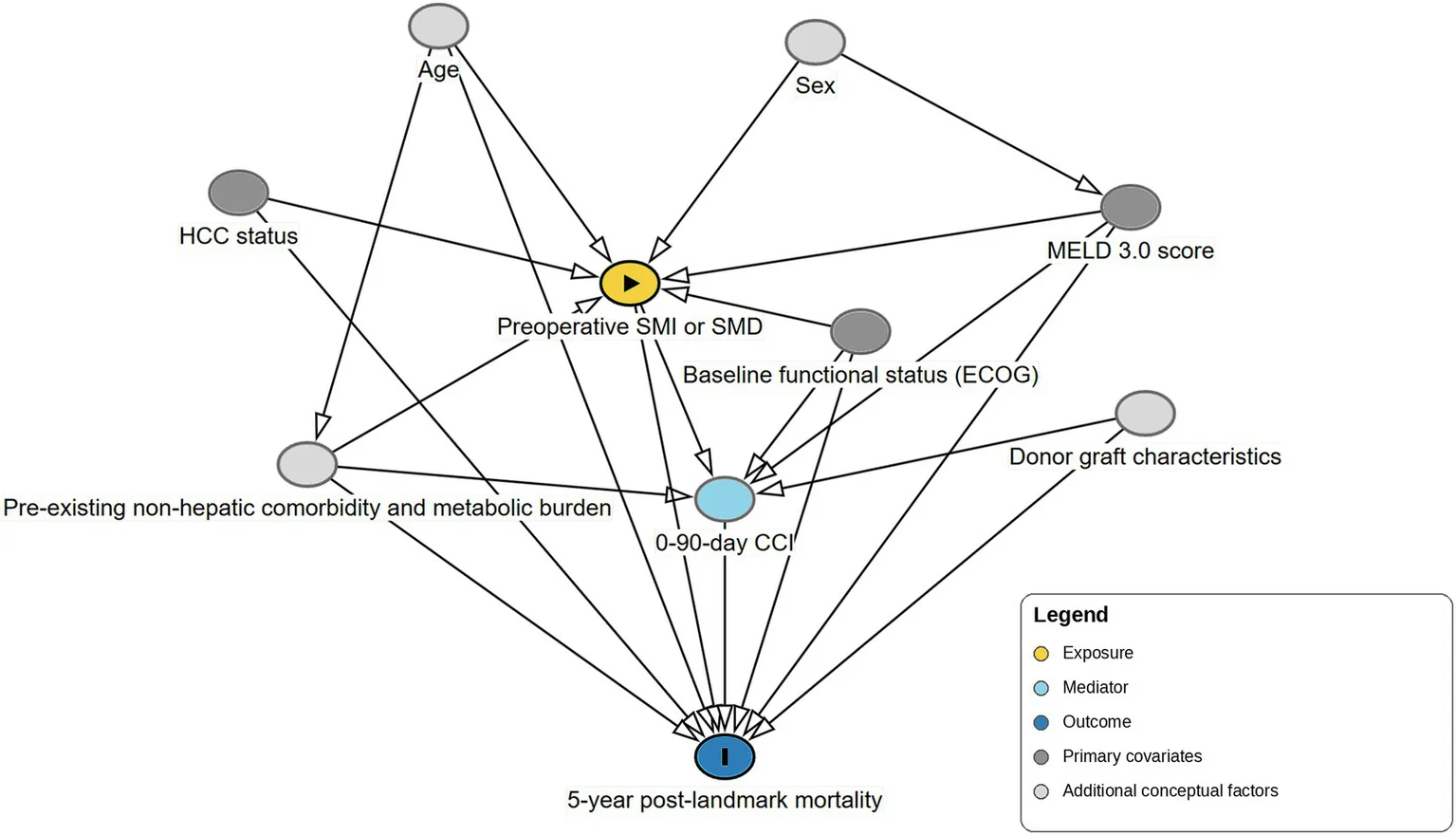
Expanded conceptual directed acyclic graph for the mediation analysis. Preoperative skeletal muscle index (SMI) and skeletal muscle density (SMD) were evaluated in separate models; the combined exposure node denotes the alternative exposure rather than a joint exposure. Cumulative Comprehensive Complication Index (CCI) accrued from transplantation to immediately before day 90 was the mediator, and all-cause mortality during the prespecified 5-year post-landmark analysis window was the outcome. Hepatocellular carcinoma (HCC) status, Eastern Cooperative Oncology Group (ECOG) performance status, and Model for End-Stage Liver Disease (MELD) 3.0 score were the prespecified measured covariates included in both models. Other depicted factors illustrate potential residual confounding and were not jointly included in the primary models. The analysis was conditional on recipients being alive and under follow-up at postoperative day 90.

### 3.2 Ninety-day postoperative complication profile

The median cumulative 90-day CCI was 43.3 (interquartile range, 32.0–52.7). At least one qualifying complication occurred in 203 recipients (99.0%), and 108 (52.7%) had a highest Clavien-Dindo grade of IIIa or higher. Infectious complications were the most frequent system category, occurring in 146 recipients (71.2%); complete distributions by highest Clavien-Dindo grade and system category are reported in Table 2. The patient-by-patient cross-check of the final adjudicated CCI records against the primary analysis database showed exact agreement for all 205 recipients (100.0%).

**Table 2:** Ninety-day postoperative complication profile and Comprehensive Complication Index data verification.

Complication characteristic	Analysis cohort (*n* = 205)
90-day Comprehensive Complication Index, median [IQR]	43.3 [32.0, 52.7]
Any postoperative complication	203 (99.0)
Major complication (highest Clavien-Dindo grade ≥IIIa)	108 (52.7)
Highest Clavien-Dindo grade	
No complication	2 (1.0)
Grade I	3 (1.5)
Grade II	92 (44.9)
Grade IIIa	63 (30.7)
Grade IIIb	14 (6.8)
Grade IVa	27 (13.2)
Grade IVb	4 (2.0)
Complication by system category	
Infectious	146 (71.2)
Hematologic and bleeding	133 (64.9)
Metabolic and renal	124 (60.5)
Graft-related	104 (50.7)
Cardiopulmonary	95 (46.3)
Wound and gastrointestinal	78 (38.0)
Neurologic	33 (16.1)
Biliary and vascular	14 (6.8)
Other	9 (4.4)
CCI data verification	
Adjudicated CCI records cross-checked, n	205
Exact concordance with the analysis database, n (%)	205 (100.0)

Data are *n* (%) unless otherwise indicated. Major complications were defined as a highest Clavien-Dindo grade of IIIa or higher. Clavien-Dindo grade V was absent because eligibility required survival to the day-90 landmark. System-category frequencies count recipients with at least one event in each category; recipients may be counted in more than one category. Exact concordance refers to agreement between the adjudicated final CCI review record and the analysis database and is not pre-adjudication inter-rater reliability. CCI, Comprehensive Complication Index; IQR, interquartile range.

### 3.3 Descriptive survival analyses

During the prespecified 5-year post-landmark analysis window, 56 of 205 recipients (27.3%) died. 67 recipients were observed alive through the full 5-year endpoint, whereas 82 were right-censored before the 5-year endpoint at their last confirmed follow-up. The median analyzed follow-up was 3.33 years (interquartile range, 1.42–5.00 years), corresponding to 634.6 person-years of analyzed follow-up. All 205 recipients contributed their observed post-landmark follow-up to the time-to-event analyses. Unadjusted Kaplan–Meier analyses compared recipients below versus at or above the cohort median (102 vs. 103 recipients for both SMI and SMD). There was no statistical evidence of a difference in overall survival for SMI (cohort median, 50.14 cm^2^/m^2^; log-rank *p* = 0.320) or SMD (cohort median, 39.49 HU; log-rank *p* = 0.298). These pooled medians were used only for descriptive visualization and were not diagnostic thresholds (Figure 3).

**Figure 3:**
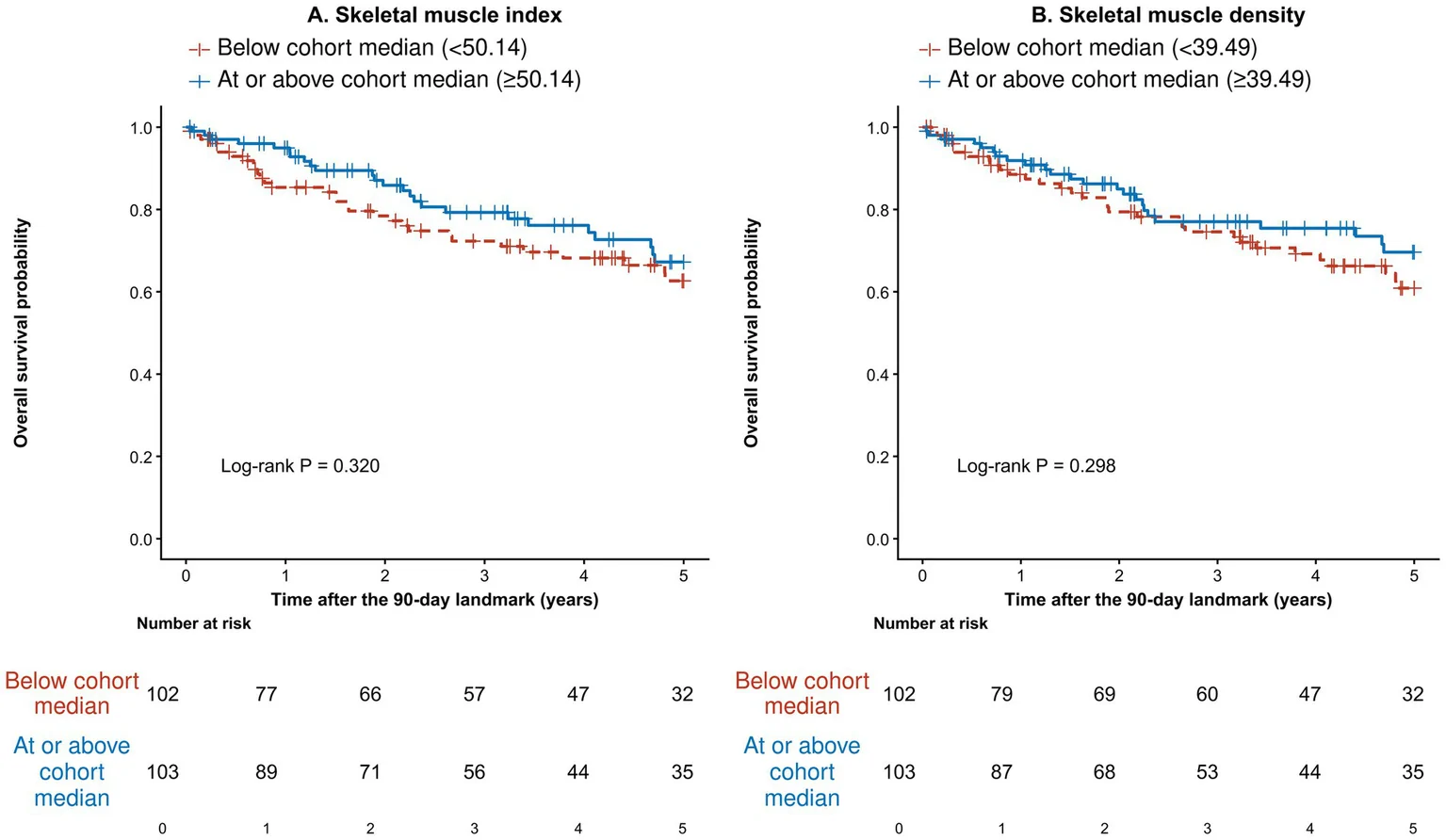
Kaplan-Meier estimates of overall survival during the prespecified 5-year post-landmark analysis window, stratified by skeletal muscle index and skeletal muscle density. **(A)** Skeletal muscle index (SMI) below the cohort median (<50.14 cm^2^/m^2^; *n* = 102) versus at or above the cohort median (≥50.14 cm^2^/m^2^; *n* = 103). **(B)** Skeletal muscle density (SMD) below the cohort median (<39.49 Hounsfield units; *n* = 102) versus at or above the cohort median (≥39.49 Hounsfield units; *n* = 103). Time zero was the postoperative day-90 landmark. Recipients were right-censored at their last confirmed follow-up before the 5-year endpoint or administratively censored at 5 years, as applicable. Tick marks indicate censoring; the tables show numbers at risk. These unadjusted analyses were descriptive, and the pooled cohort medians were not diagnostic thresholds. Log-rank *p* values were 0.320 for SMI and 0.298 for SMD.

### 3.4 Primary mediation estimates and E-value analysis

The prespecified natural-effect contrasts were estimated separately for SMI and SMD in relation to all-cause mortality during the prespecified 5-year post-landmark analysis window, with cumulative 90-day CCI as the mediator. Both the mediator and outcome models included the prespecified measured covariates of HCC status, ECOG performance status, and MELD 3.0 score. Effects associated with a 1-standard deviation decrease in each exposure are reported as approximate effects on the HR scale. For SMI, the HRs were 1.155 (95% bootstrap CI, 1.033–1.326) for the NIE, 1.155 (0.869–1.634) for the NDE, and 1.335 (1.026–1.902) for the TE. The corresponding HRs for SMD were 1.190 (1.062–1.382), 0.987 (0.710–1.393), and 1.175 (0.881–1.608), respectively (Table 3). The percentile-bootstrap 95% CIs did not include 1 for either NIE or for the SMI TE, whereas the CIs for both NDEs and the SMD TE included 1.

**Table 3:** Primary mediation estimates through the 90-day Comprehensive Complication Index.

Exposure contrast	Effect	Measure	Estimate (95% bootstrap CI)	NIE E-value (point; CI limit)
SMI, 1-SD decrease	Natural indirect effect	Hazard ratio	1.155 (1.033–1.326)	1.579; 1.217
SMI, 1-SD decrease	Natural direct effect	Hazard ratio	1.155 (0.869–1.634)	–
SMI, 1-SD decrease	Total effect	Hazard ratio	1.335 (1.026–1.902)	–
SMD, 1-SD decrease	Natural indirect effect	Hazard ratio	1.190 (1.062–1.382)	1.666; 1.317
SMD, 1-SD decrease	Natural direct effect	Hazard ratio	0.987 (0.710–1.393)	–
SMD, 1-SD decrease	Total effect	Hazard ratio	1.175 (0.881–1.608)	–

Natural indirect, natural direct, and total effects are HR-scale approximate effects for all-cause mortality during the prespecified 5-year post-landmark analysis window. Confidence intervals are percentile-bootstrap 95% CIs based on 1,000 nonparametric resamples. CCI was the mediator; models included HCC status, ECOG performance status, and MELD 3.0. E-values are exploratory and are reported only for the NIEs as the point-estimate E-value and the E-value for the confidence limit closest to the null. For E-value calculation, NIE HRs were treated as approximate risk ratios. CCI, Comprehensive Complication Index; CI, confidence interval; ECOG, Eastern Cooperative Oncology Group; HCC, hepatocellular carcinoma; HR, hazard ratio; MELD, Model for End-Stage Liver Disease; NIE, natural indirect effect; SD, standard deviation; SMD, skeletal muscle density; SMI, skeletal muscle index.

In the exploratory analysis treating the NIE HRs as approximate risk ratios, the mediational E-values for the NIE point estimates and the corresponding confidence limits closest to the null value of 1 were 1.58 and 1.22 for SMI and 1.67 and 1.32 for SMD, respectively (Supplementary Figure S1). The primary mediation estimates are displayed in Figure 4.

**Figure 4:**
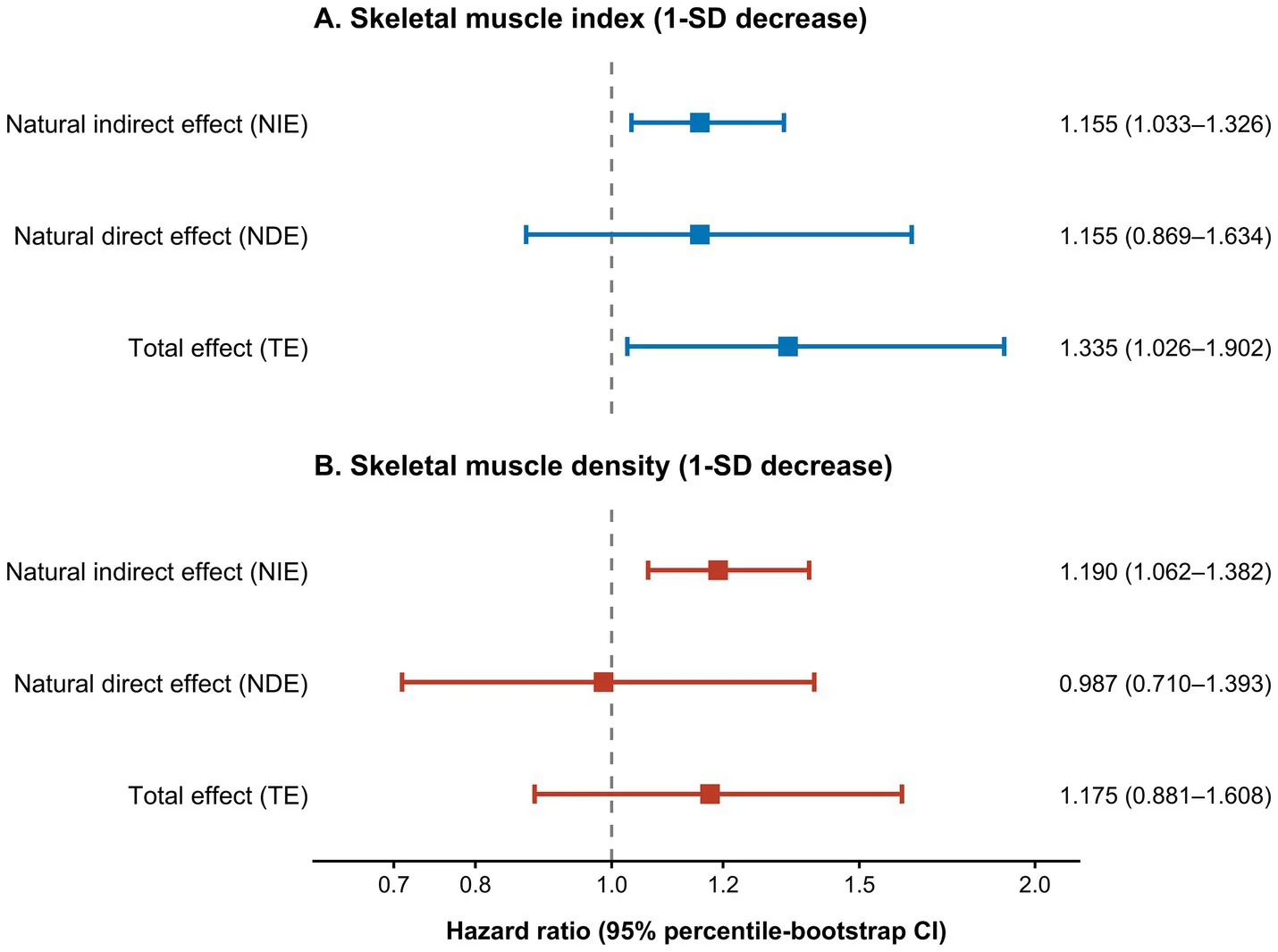
Natural indirect, natural direct, and total effects of skeletal muscle index and skeletal muscle density on all-cause mortality during the prespecified 5-year post-landmark analysis window. **(A)** Effects associated with a 1-standard deviation decrease in skeletal muscle index (SMI). **(B)** Corresponding effects associated with a 1-standard deviation decrease in skeletal muscle density (SMD). Cumulative Comprehensive Complication Index accrued from completion of liver transplantation to immediately before the postoperative day-90 landmark was the mediator. Models were adjusted for hepatocellular carcinoma, Eastern Cooperative Oncology Group performance status, and Model for End-Stage Liver Disease 3.0 score. Squares are hazard-ratio estimates; horizontal lines denote percentile-based 95% confidence intervals obtained from 1,000 nonparametric bootstrap resamples. The dashed vertical line denotes the null value of 1. CI, confidence interval; NDE, natural direct effect; NIE, natural indirect effect; TE, total effect.

### 3.5 Robustness and sensitivity analyses

Replacing ECOG performance status with age yielded NIE HRs of 1.160 (95% CI, 1.037–1.324) for SMI and 1.189 (1.064–1.382) for SMD, similar to the primary estimates. There was no statistical evidence of exposure-by-CCI interaction in the outcome models (likelihood-ratio *p* = 0.823 for SMI and 0.155 for SMD). After CCI was dichotomized at the 75th percentile (>52.7; 51 high-CCI and 154 non-high-CCI recipients), the NIE HRs were attenuated to 1.114 (0.992–1.379) and 1.140 (1.020–1.373), respectively; the CI included 1 for SMI but not for SMD (Supplementary Table S2).

Within the HCC subgroup (*n* = 113), SMI and SMD were re-standardized within the subgroup, and the NIE HRs were 1.089 (95% CI, 0.952–1.320) and 1.148 (0.993–1.400), respectively. Both were directionally consistent with the primary analysis, although both CIs included 1 (Supplementary Table S3). In an additional exploratory analysis, the exposure-by-HCC-status interaction *p* values for the associations with 90-day CCI were 0.408 for SMI and 0.218 for SMD. Neither test provided statistical evidence of effect modification by HCC status (Supplementary Table S3). Exploratory analyses showed no statistical evidence of effect modification by sex (Supplementary Table S6). The delayed day-120 landmark analysis included 200 recipients and 54 deaths and yielded NIE HRs of 1.155 (1.035–1.317) for SMI and 1.187 (1.056–1.364) for SMD (Supplementary Table S4). Cumulative CCI from completion of transplantation to immediately before day 90 remained the mediator; only outcome follow-up was delayed to day 120. Study-specific identification assumptions and related concerns are summarized in Supplementary Table S5.

Using the external sex-specific FLEXIT/Carey thresholds, 86 of 205 recipients (42.0%; 83 men and 3 women) were classified as having low SMI. Low SMI was associated with a higher adjusted mean 90-day CCI (difference, 7.900; 95% CI, 3.731–12.068; *p* < 0.001), but no statistical evidence of an association with 5-year post-landmark mortality was detected (adjusted HR, 1.293; 95% CI, 0.727–2.298; *p* = 0.382; log-rank *p* = 0.734; Supplementary Table S7).

Proportional-hazards diagnostics showed no statistical evidence of violation of the proportional hazards assumption, with global *p* values of 0.544 and 0.603 for the SMI and SMD outcome models, respectively (Supplementary Figure S2). The mediator models showed no clear evidence of heteroscedasticity. There was no statistical evidence of departure from residual normality for the SMI mediator model, whereas the SMD mediator model showed a mild departure from normality (Shapiro–Wilk *p* = 0.044; Supplementary Figure S3). The bootstrap NIE distributions are shown in Supplementary Figure S4.

## 4 Discussion

This study evaluated whether cumulative 90-day postoperative complication burden was compatible with an intermediate pathway linking preoperative CT-derived skeletal muscle phenotypes to mortality during a prespecified 5-year post-landmark analysis window. In separate models including HCC status, ECOG performance status, and MELD 3.0 score as the prespecified measured covariates, lower SMI and SMD showed model-based approximate NIEs on the HR scale through cumulative CCI. For SMI, the CIs for the NIE and TE did not include 1, whereas the NDE CI included 1; for SMD, only the NIE CI did not include 1. NIE estimates were similar after covariate substitution and delayed outcome follow-up to day 120 but attenuated after CCI dichotomization. These findings are compatible with postoperative complication burden as a potential intermediate clinical pathway, but they do not establish a mechanism.

The descriptive Kaplan–Meier and mediation analyses addressed different estimands. The former used unadjusted groups defined by pooled cohort medians solely for descriptive visualization; these medians were not clinical or diagnostic cutoffs. The latter retained continuous standardized exposures and separated model-based indirect and direct-effect contrasts. Dichotomization facilitates presentation but discards information and may reduce statistical power (26). The landmark design ensured that the prespecified CCI ascertainment window ended before outcome follow-up began, preserving the intended temporal ordering between mediator assessment and the post-landmark risk period. Nevertheless, the post-landmark estimates are conditional on survival and continued follow-up through postoperative day 90 and do not apply to recipients excluded before the landmark (14).

Lower SMI reflects lower muscle area relative to height and may indicate reduced muscle reserve. In cirrhosis, hyperammonemia, inflammation, inadequate intake, and disrupted amino-acid metabolism can promote muscle catabolism (3, 4, 27), potentially impairing immune defense, tissue repair, and functional recovery after transplantation. This interpretation is consistent with evidence linking low CT-assessed muscle mass to adverse outcomes among liver transplantation candidates and recipients (28). The observed model-based SMI NIE is compatible with postoperative complication burden representing one intermediate clinical pathway, but the present data cannot distinguish the contributions of altered protein metabolism, impaired infection defense, or compromised tissue repair.

Compared with SMI, which primarily reflects muscle quantity, SMD reflects the mean attenuation of segmented skeletal muscle and is commonly interpreted as an imaging marker of muscle composition. Lower CT attenuation is associated with myosteatosis (29), although SMD does not directly quantify microscopic fat infiltration. Proposed mechanisms include abnormal lipid accumulation, insulin resistance, mitochondrial dysfunction, and impaired muscle metabolism (30), and previous transplantation cohorts have associated myosteatosis with greater perioperative burden and poorer outcomes (5, 31). In this study, the SMD NIE CI did not include 1, whereas the NDE estimate was close to 1 and the TE CI included 1. This pattern is compatible with postoperative complication burden participating in the pathway linking lower SMD to long-term outcomes, but it does not establish CCI as the predominant or complete mediator.

By integrating the number and severity of complications, CCI describes cumulative postoperative burden more comprehensively than the single highest Clavien-Dindo grade (9). Previous liver transplantation studies have associated higher CCI with short- and long-term adverse outcomes (10, 11). CCI may therefore serve as an integrated clinical phenotype of the postoperative course. Nevertheless, it is jointly shaped by recipient and donor characteristics, operative events, and treatment and cannot be interpreted as a single biological mechanism. Because it is a composite mediator, this study could not determine the relative contributions of individual complication categories to the observed indirect effects.

The similar NIE estimates after replacing ECOG performance status with age and after delaying outcome follow-up to day 120 suggest some stability to these analytical choices. There was no statistical evidence of exposure-by-CCI interaction in the outcome models. Dichotomizing CCI attenuated both NIEs, with the SMI CI including 1 but the SMD CI not including 1. Within the HCC subgroup, both NIE CIs included 1 despite directions consistent with the primary analysis; reduced sample size, fewer events, and differences in patient composition may all have contributed. Exploratory interaction analyses similarly showed no statistical evidence that the association of either SMI or SMD with 90-day CCI differed by HCC status. Because HCC status is not a complete classification of underlying liver-disease etiology, these analyses do not address differences among specific etiologies. The lack of statistical evidence for sex interactions should likewise be interpreted cautiously because interaction tests had limited power (32). The external-threshold analysis classified low SMI in only three women and showed a higher adjusted 90-day CCI but no statistical evidence of a mortality association; its interpretation is limited by dichotomization and the marked sex-specific imbalance. Proportional-hazards diagnostics showed no statistical evidence of nonproportionality, and heteroscedasticity assessments showed no statistical evidence of heteroscedasticity; however, the SMD mediator model showed a mild departure from residual normality. These diagnostics should be interpreted as model checks rather than proof that the underlying assumptions hold.

The clinical-nutrition relevance of these findings lies in complementary characterization of preoperative body-composition vulnerability rather than evidence of an intervention effect. When preoperative CT is already available, SMI and SMD may provide opportunistic information on body-composition vulnerability and may complement comprehensive clinical, nutritional, and functional or frailty assessment (1). They should not replace validated nutritional assessment, muscle-strength testing, or physical-function assessment, and neither SMI nor SMD alone establishes a diagnosis of malnutrition or sarcopenia. Pilot randomized research and a recent systematic review and meta-analysis suggest that pretransplant prehabilitation is feasible and may improve selected activity or physical-function measures, but evidence that it reduces post-transplant CCI or long-term mortality remains insufficient (33, 34). Whether CT-derived muscle assessment can guide effective nutritional or multimodal prehabilitation strategies requires prospective multicenter evaluation.

Strengths of this study include explicit temporal ordering of preoperative CT measurements, CCI ascertainment before the day-90 landmark, and post-landmark survival; blinded independent physician review of postoperative complications with adjudication and patient-by-patient verification of the final CCI records; and evaluation of sensitivity through nonparametric bootstrap resampling, alternative model specifications, delayed outcome follow-up, and model diagnostics.

Several limitations warrant emphasis. First, this was a single-center retrospective study, and the landmark cohort included only recipients alive and under follow-up on postoperative day 90; the post-landmark estimates therefore do not apply to recipients who died before day 90 or whose recorded follow-up ended before the landmark. The cohort comprised 205 recipients with 56 deaths during the prespecified 5-year post-landmark analysis window, limiting the precision of complex models, subgroup analyses, and interaction tests. In addition, 82 recipients were right-censored before the 5-year endpoint, reducing the amount of information available toward the later part of the analysis window, although all contributed their observed follow-up time to the survival analyses. Much of this shorter follow-up reflected the available calendar time for more recent transplantations; nevertheless, informative censoring cannot be completely excluded. Moreover, the 27.3% mortality rate was not strictly rare, so the Cox-based decomposition should be interpreted as approximate mediation effects on the HR scale rather than exact causal effects on the risk-ratio scale.

Second, the expanded conceptual DAG indicates that age, pre-existing non-hepatic comorbidity and metabolic burden, donor graft characteristics, and other factors may affect relevant pathways. The primary models included HCC status, ECOG performance status, and MELD 3.0 score, but these prespecified measured covariates cannot be assumed to account for all potential confounding pathways. Residual and unmeasured confounding, including factors related to the operative course, immunosuppression, nutritional status, systemic inflammation, and donor quality, may affect relevant exposure-mediator-outcome relationships. Strict causal interpretation therefore depends on identification assumptions that cannot be fully verified; the E-values quantify only the association strength required for selected unmeasured mediator-outcome confounding scenarios and do not establish the other identification assumptions.

Finally, detailed underlying liver-disease etiologies were unavailable, precluding formal etiology-stratified analyses; the HCC-status analysis should therefore be interpreted only as a limited broad-category comparison. The study also did not directly measure dietary intake, validated nutritional-screening scores, muscle strength, or physical performance. A single preoperative CT cannot capture longitudinal changes in muscle status, and formal inter- and intra-reader reliability analyses were not performed. Neither SMI nor SMD alone establishes a diagnosis of malnutrition or sarcopenia, and the composite CCI cannot identify category-specific mediation. Multicenter prospective cohorts incorporating repeated body-composition assessment, comprehensive nutritional evaluation, and functional measures are needed to confirm these findings and evaluate their clinical relevance.

## 5 Conclusion

Among liver transplant recipients alive and under follow-up through postoperative day 90, lower preoperative SMI and SMD showed model-based approximate natural indirect effects on the HR scale through cumulative postoperative CCI. These CT-derived muscle phenotypes may complement, but should not replace, comprehensive clinical, nutritional, and functional assessment, and the findings are compatible with postoperative complication burden as a potential intermediate clinical pathway. However, the observational design, non-rare outcome, and incompletely verifiable identification assumptions preclude strict causal or interventional interpretation. Prospective multicenter studies incorporating longitudinal body-composition, nutritional, and functional assessment are needed to confirm these findings and determine their clinical utility.

## Data Availability

The original contributions presented in the study are included in the article/Supplementary material, further inquiries can be directed to the corresponding author.

## References

[1] Lai JC Sonnenday CJ Tapper EB Duarte-Rojo A Dunn MA Bernal W . Frailty in liver transplantation: an expert opinion statement from the American Society of Transplantation liver and intestinal Community of Practice. Am J Transplant. (2019) 19:1896–906. doi: 10.1111/ajt.15392, 30980701 PMC6814290

[2] Lai JC Ufere NN Bucuvalas JC. Liver transplant survivorship. Liver Transpl. (2020) 26:1030–3. doi: 10.1002/lt.25792, 32372563 PMC7384890

[3] Dasarathy S Merli M. Sarcopenia from mechanism to diagnosis and treatment in liver disease. J Hepatol. (2016) 65:1232–44. doi: 10.1016/j.jhep.2016.07.040, 27515775 PMC5116259

[4] Ebadi M Bhanji RA Mazurak VC Montano-Loza AJ. Sarcopenia in cirrhosis: from pathogenesis to interventions. J Gastroenterol. (2019) 54:845–59. doi: 10.1007/s00535-019-01605-6, 31392488 PMC6759678

[5] Czigany Z Kramp W Bednarsch J van der Kroft G Boecker J Strnad P . Myosteatosis to predict inferior perioperative outcome in patients undergoing orthotopic liver transplantation. Am J Transplant. (2020) 20:493–503. doi: 10.1111/ajt.15577, 31448486

[6] Cederholm T Jensen GL Correia MITD Gonzalez MC Fukushima R Higashiguchi T . GLIM criteria for the diagnosis of malnutrition: a consensus report from the global clinical nutrition community. Clin Nutr. (2019) 38:1–9. doi: 10.1016/j.clnu.2018.08.002, 30181091

[7] Cruz-Jentoft AJ Bahat G Bauer J Boirie Y Bruyère O Cederholm T . Sarcopenia: revised European consensus on definition and diagnosis. Age Ageing. (2019) 48:16–31. doi: 10.1093/ageing/afy169, 30312372 PMC6322506

[8] Markakis GE Lai JC Karakousis ND Papatheodoridis GV Psaltopoulou T Merli M . Sarcopenia as a predictor of survival and complications of patients with cirrhosis after liver transplantation: a systematic review and meta-analysis. Clin Transpl. (2025) 39:e70088. doi: 10.1111/ctr.70088, 39876624 PMC11775496

[9] Slankamenac K Graf R Barkun J Puhan MA Clavien PA. The comprehensive complication index: a novel continuous scale to measure surgical morbidity. Ann Surg. (2013) 258:1–7. doi: 10.1097/SLA.0b013e318296c732, 23728278

[10] Lai Q Melandro F Nowak G Nicolini D Iesari S Fasolo E . The role of the comprehensive complication index for the prediction of survival after liver transplantation. Updat Surg. (2021) 73:209–21. doi: 10.1007/s13304-020-00878-4, 32892294 PMC7889667

[11] Castanedo S Toledo E Fernández-Santiago R Castillo F Echeverri J Rodríguez-Sanjuán JC. Influence of postoperative complications on long-term survival in liver transplant patients. World J Gastrointest Surg. (2020) 12:336–45. doi: 10.4240/wjgs.v12.i8.336, 32903918 PMC7448209

[12] VanderWeele TJ. Mediation analysis: a practitioner’s guide. Annu Rev Public Health. (2016) 37:17–32. doi: 10.1146/annurev-publhealth-032315-021402, 26653405

[13] Anderson JR Cain KC Gelber RD. Analysis of survival by tumor response. J Clin Oncol. (1983) 1:710–9. doi: 10.1200/JCO.1983.1.11.710, 6668489

[14] Gleiss A Oberbauer R Heinze G. An unjustified benefit: immortal time bias in the analysis of time-dependent events. Transpl Int. (2018) 31:125–30. doi: 10.1111/tri.13081, 29024071

[15] Dindo D Demartines N Clavien PA. Classification of surgical complications: a new proposal with evaluation in a cohort of 6336 patients and results of a survey. Ann Surg. (2004) 240:205–13. doi: 10.1097/01.SLA.0000133083.54934.AE, 15273542 PMC1360123

[16] Greenland S Pearl J Robins JM. Causal diagrams for epidemiologic research. Epidemiology. (1999) 10:37–48. doi: 10.1097/00001648-199901000-00008, 9888278

[17] Kim WR Mannalithara A Heimbach JK Kamath PS Asrani SK Biggins SW . MELD 3.0: the model for end-stage liver disease updated for the modern era. Gastroenterology. (2021) 161:1887–1895.e4. doi: 10.1053/j.gastro.2021.08.050, 34481845 PMC8608337

[18] VanderWeele TJ. Causal mediation analysis with survival data. Epidemiology. (2011) 22:582–5. doi: 10.1097/EDE.0b013e31821db37e, 21642779 PMC3109321

[19] Valeri L VanderWeele TJ. Mediation analysis allowing for exposure-mediator interactions and causal interpretation: theoretical assumptions and implementation with SAS and SPSS macros. Psychol Methods. (2013) 18:137–50. doi: 10.1037/a0031034, 23379553 PMC3659198

[20] VanderWeele TJ Ding P. Sensitivity analysis in observational research: introducing the E-value. Ann Intern Med. (2017) 167:268–74. doi: 10.7326/M16-2607, 28693043

[21] Smith LH VanderWeele TJ. Mediational E-values: approximate sensitivity analysis for unmeasured mediator-outcome confounding. Epidemiology. (2019) 30:835–7. doi: 10.1097/EDE.0000000000001064, 31348008 PMC6768718

[22] Grambsch PM Therneau TM. Proportional hazards tests and diagnostics based on weighted residuals. Biometrika. (1994) 81:515–26. doi: 10.1093/biomet/81.3.515

[23] World Medical Association. World Medical Association declaration of Helsinki: ethical principles for medical research involving human participants. JAMA. (2025) 333:71–4. doi: 10.1001/jama.2024.21972, 39425955

[24] The declaration of Istanbul on organ trafficking and transplant tourism (2018 edition). Transplantation. (2019) 103:218–9. doi: 10.1097/TP.0000000000002540 30681644

[25] Huang J Wang H Fan ST Zhao B Zhang Z Hao L . The national program for deceased organ donation in China. Transplantation. (2013) 96:5–9. doi: 10.1097/TP.0b013e3182985491, 23743728

[26] Altman DG Royston P. The cost of dichotomising continuous variables. BMJ. (2006) 332:1080. doi: 10.1136/bmj.332.7549.1080, 16675816 PMC1458573

[27] Davuluri G Krokowski D Guan BJ Kumar A Thapaliya S Singh D . Metabolic adaptation of skeletal muscle to hyperammonemia drives the beneficial effects of L-leucine in cirrhosis. J Hepatol. (2016) 65:929–37. doi: 10.1016/j.jhep.2016.06.004, 27318325 PMC5069194

[28] van Vugt JLA Levolger S de Bruin RWF van Rosmalen J Metselaar HJ IJzermans JNM. Systematic review and meta-analysis of the impact of computed tomography-assessed skeletal muscle mass on outcome in patients awaiting or undergoing liver transplantation. Am J Transplant. (2016) 16:2277–92. doi: 10.1111/ajt.13732 26813115

[29] Aubrey J Esfandiari N Baracos VE Buteau FA Frenette J Putman CT . Measurement of skeletal muscle radiation attenuation and basis of its biological variation. Acta Physiol (Oxf). (2014) 210:489–97. doi: 10.1111/apha.12224, 24393306 PMC4309522

[30] Ebadi M Tsien C Bhanji RA Dunichand-Hoedl AR Rider E Motamedrad M . Myosteatosis in cirrhosis: a review of diagnosis, pathophysiological mechanisms and potential interventions. Cells. (2022) 11:1216. doi: 10.3390/cells11071216, 35406780 PMC8997850

[31] Czigany Z Kramp W Lurje I Miller H Bednarsch J Lang SA . The role of recipient myosteatosis in graft and patient survival after deceased donor liver transplantation. J Cachexia Sarcopenia Muscle. (2021) 12:358–67. doi: 10.1002/jcsm.12669, 33525056 PMC8061365

[32] Sun X Ioannidis JPA Agoritsas T Alba AC Guyatt G. How to use a subgroup analysis: users’ guide to the medical literature. JAMA. (2014) 311:405–11. doi: 10.1001/jama.2013.285063, 24449319

[33] Serper M Jones LS Clement T Reddy KR Reese PP. A randomized, controlled, prehabilitation intervention to maximize early recovery (PRIMER) in liver transplantation. Liver Transpl. (2024) 30:10–9. doi: 10.1097/LVT.0000000000000198, 37379030 PMC10755068

[34] Abow-Mohamed I Martel M Janaudis-Ferreira T Gillis C Geraci O Albader F . Feasibility, safety, and effectiveness of prehabilitation programs in patients awaiting liver transplantation: a systematic review and meta-analysis. Am J Transplant. (2026) 26:1110–20. doi: 10.1016/j.ajt.2026.01.007, 41654085

