# Supplementary material for: Preoperative CT-derived skeletal muscle phenotypes, postoperative complication burden, and long-term survival after liver transplantation: a 90-day landmark mediation analysis
Source: Front Nutr. 2026 Sep 28;13:1978429. doi: 10.3389/fnut.2026.1978429 (PMC13654752; doi:10.3389/fnut.2026.1978429)
Supplement: Supplementary file 1 [file Data_Sheet_1.docx]

STROBE Statement—Checklist for the manuscript

Page and line references in this checklist refer to the accepted manuscript before typesetting.

| **Section / topic** | **Item No.** | **Recommendation** | **Page(s) / location** |
| --- | --- | --- | --- |
| Title and abstract | 1(a) | Indicate the study’s design with a commonly used term in the title or the abstract. | p. 1, lines 2–5 and 21–46 (title and structured Abstract); p. 2, lines 101–113 (Section 2.1: single-center retrospective cohort and day-90 landmark). |
| Title and abstract | 1(b) | Provide in the abstract an informative and balanced summary of what was done and what was found. | p. 1, lines 21–46 (structured Abstract: Background, Methods, Results, and Conclusion). |
| Background/rationale | 2 | Explain the scientific background and rationale for the investigation being reported. | pp. 1–2, lines 50–93 (Introduction: scientific background, CT-derived muscle phenotypes, diagnostic context, CCI, and rationale for an intermediate pathway). |
| Objectives | 3 | State specific objectives, including any prespecified hypotheses. | p. 2, lines 94–100 (Introduction, final paragraph: objective and prespecified hypotheses). |
| Study design | 4 | Present key elements of study design early in the paper. | p. 2, lines 101–113 (Section 2.1); pp. 3–4, lines 160–180 (conceptual framework and covariate specification); p. 12, lines 608–627 (Figures 1–2 legends). |
| Setting | 5 | Describe the setting, locations, and relevant dates, including periods of recruitment, exposure, follow-up, and data collection. | p. 2, lines 103–107 (single-center setting, Fuzhou, China; January 2016–December 2025); p. 3, lines 148–159 (follow-up window, sources, and database lock on March 31, 2026). |
| Participants | 6(a) | Give the eligibility criteria, and the sources and methods of selection of participants. Describe methods of follow-up. | pp. 2–3, lines 103–113 and 148–159 (eligibility, landmark construction, follow-up sources, and censoring); p. 12, lines 608–617 (Figure 1 legend). |
| Participants | 6(b) | For matched studies, give matching criteria and number of exposed and unexposed. | Not applicable (unmatched cohort). |
| Variables | 7 | Clearly define all outcomes, exposures, predictors, potential confounders, and effect modifiers. Give diagnostic criteria, if applicable. | pp. 2–5, lines 60–80, 114–180, and 221–238 (GLIM/EWGSOP2 diagnostic context; SMI/SMD exposures; CCI mediator; mortality outcome; measured covariates; and exploratory effect modifiers); Supplementary Methods Sections 1–3.6. |
| Data sources/measurement | 8* | For each variable of interest, give sources of data and details of methods of assessment (measurement). Describe comparability of assessment methods if there is more than one group. | p. 3, lines 114–159 (CT measurement, CCI ascertainment/adjudication, and death and last-follow-up sources); Supplementary Methods Sections 1.1–1.2. |
| Bias | 9 | Describe any efforts to address potential sources of bias. | pp. 2–5, lines 108–113, 141–147, 160–180, 190–195, and 208–238 (landmarking, blinded CCI review, DAG/confounding framework, landmark comparison, and sensitivity analyses); pp. 8–9, lines 449–482 (limitations); Supplementary Table S5. |
| Study size | 10 | Explain how the study size was arrived at. | p. 2, lines 103–113; p. 4, lines 181–189. Consecutive eligible recipients were screened; no a priori sample-size calculation was performed. |
| Quantitative variables | 11 | Explain how quantitative variables were handled in the analyses. If applicable, describe which groupings were chosen and why. | pp. 3–5, lines 124–130 and 182–238. SMI/SMD were standardized with a prespecified 1-SD decrease; pooled-median dichotomization was descriptive; CCI dichotomization and external sex-specific low-SMI thresholds were sensitivity analyses. |
| Statistical methods | 12(a) | Describe all statistical methods, including those used to control for confounding. | pp. 3–5, lines 160–245 (covariate specification; descriptive statistics; KM/log-rank; OLS mediator and Cox outcome models; mediation estimands; bootstrap CIs; E-values; robustness analyses; and diagnostics); Supplementary Methods Sections 2–3.6. |
| Statistical methods | 12(b) | Describe any methods used to examine subgroups and interactions. | pp. 4–5, lines 221–238 (exposure×CCI interaction, HCC subgroup, sex and HCC effect modification, and external-threshold low-SMI analysis); Supplementary Methods Sections 3.2 and 3.6; Supplementary Tables S3, S6, and S7. |
| Statistical methods | 12(c) | Explain how missing data were addressed. | p. 4, lines 182–189. Primary-analysis variables had no missing data; no imputation was required. |
| Statistical methods | 12(d) | If applicable, explain how loss to follow-up was addressed. | p. 3, lines 148–159 (censoring rules); p. 6, lines 300–305 (follow-up disposition); p. 12, lines 608–617 (Figure 1 legend). |
| Statistical methods | 12(e) | Describe any sensitivity analyses. | pp. 4–5, lines 221–245; pp. 6–7, lines 328–361; Supplementary Methods Sections 3.2–3.6; Supplementary Tables S2–S7 and Figures S1–S4. |
| Participants | 13(a)* | Report numbers of individuals at each stage of study—eg numbers potentially eligible, examined for eligibility, confirmed eligible, included in the study, completing follow-up, and analysed. | p. 5, lines 273–290; p. 6, lines 300–305; p. 12, lines 608–617 (Figure 1). Flow: 245 screened → 233 baseline eligible → 205 landmark cohort; 56 deaths, 67 alive through 5 years, and 82 right-censored before 5 years. |
| Participants | 13(b)* | Give reasons for non-participation at each stage. | p. 5, lines 273–278; p. 12, lines 608–617. Initial exclusions: inadequate preoperative CT data or unavailable key clinical data; pre-landmark exclusions: 25 deaths and 3 with recorded follow-up ending before day 90. |
| Participants | 13(c)* | Consider use of a flow diagram. | p. 12, lines 608–617 (Figure 1 and legend). |
| Descriptive data | 14(a)* | Give characteristics of study participants (eg demographic, clinical, social) and information on exposures and potential confounders. | p. 5, lines 280–290; p. 13, Table 1 (demographic, body-composition, oncologic, liver-disease severity, and functional-status characteristics); Supplementary Table S6 (sex-specific SMI/SMD distributions). |
| Descriptive data | 14(b)* | Indicate number of participants with missing data for each variable of interest. | p. 4, lines 182–189. Primary-analysis variables had no missing data in the final 205-recipient landmark cohort. |
| Descriptive data | 14(c)* | Summarise follow-up time (eg, average and total amount). | p. 6, lines 300–305. Median analyzed follow-up was 3.33 years (IQR, 1.42–5.00), totaling 634.6 person-years. |
| Outcome data | 15* | Report numbers of outcome events or summary measures over time. | p. 6, lines 300–311 (56 deaths and descriptive survival results); p. 12, lines 628–637 (Figure 3 legend). |
| Main results | 16(a) | Give unadjusted estimates and, if applicable, confounder-adjusted estimates and their precision (eg, 95% confidence interval). Make clear which confounders were adjusted for and why they were included. | pp. 3–4, lines 160–214 (measured-covariate rationale and model specification); p. 6, lines 300–327 (unadjusted KM and adjusted mediation results); p. 12, lines 628–648 (Figures 3–4 legends); p. 14, Table 3, lines 662–670. |
| Main results | 16(b) | Report category boundaries when continuous variables were categorized. | pp. 4–5, lines 196–200 and 221–238; pp. 6–7, lines 307–311 and 333–354; p. 12, lines 628–637 (SMI median 50.14 cm²/m²; SMD median 39.49 HU; high CCI >52.7; FLEXIT/Carey low SMI: men <50 and women <39 cm²/m²); Supplementary Tables S2 and S7. |
| Main results | 16(c) | If relevant, consider translating estimates of relative risk into absolute risk for a meaningful time period. | Not applicable to the prespecified HR-scale approximate natural-effect estimands; the observed 5-year post-landmark mortality proportion is reported separately. |
| Other analyses | 17 | Report other analyses done—eg analyses of subgroups and interactions, and sensitivity analyses. | pp. 6–7, lines 328–361; Supplementary Tables S2–S7 and Figures S1–S4 (subgroup, interaction, robustness, sensitivity, and diagnostic analyses). |
| Key results | 18 | Summarise key results with reference to study objectives. | p. 7, lines 362–373 (Discussion opening paragraph). |
| Limitations | 19 | Discuss limitations of the study, taking into account sources of potential bias or imprecision. Discuss both direction and magnitude of any potential bias. | pp. 8–9, lines 449–482 (single-center design, landmark conditioning, limited events, pre-5-year censoring/informative censoring, non-rare outcome, residual confounding, unavailable detailed etiologies, nutritional/functional measurement limitations, and imaging reliability); Supplementary Table S5. |
| Interpretation | 20 | Give a cautious overall interpretation of results considering objectives, limitations, multiplicity of analyses, results from similar studies, and other relevant evidence. | pp. 7–9, lines 362–493 (Discussion and Conclusion: cautious interpretation, relation to prior evidence, alternative estimands, sensitivity analyses, Clinical Nutrition relevance, and causal/interventional limitations). |
| Generalisability | 21 | Discuss the generalisability (external validity) of the study results. | pp. 8–9, lines 449–493 (single-center and landmark-selected population; need for prospective multicenter cohorts incorporating longitudinal body-composition, nutritional, and functional assessment). |
| Funding | 22 | Give the source of funding and the role of the funders for the present study and, if applicable, for the original study on which the present article is based. | p. 9, lines 509–511 (Funding: no financial support was received for the research and/or publication). |

**Give information separately for exposed and unexposed groups where applicable.*
