# Supplementary material for: Preoperative CT-derived skeletal muscle phenotypes, postoperative complication burden, and long-term survival after liver transplantation: a 90-day landmark mediation analysis
Source: Front Nutr. 2026 Sep 28;13:1978429. doi: 10.3389/fnut.2026.1978429 (PMC13654752; doi:10.3389/fnut.2026.1978429)
Supplement: Supplementary file 2 [file Data_Sheet_2.docx]

**Supplementary Materials**

**Section 1: Clinical Definitions and Measurement Protocols**

**1.1 Skeletal Muscle CT Measurement and Feature Extraction**

This section details the standard operating procedures for baseline measurement and quality control of preoperative skeletal muscle index (SMI) and skeletal muscle density (SMD).

**1.1.1 Image Selection and Anatomical Localization**

The most recent routine abdominal computed tomography (CT) scan acquired within 3 months before liver transplantation was retrieved. On the axial series, a single image at the mid-level of the third lumbar vertebra (L3) was selected, defined as the level at which both transverse processes were fully visible and the spinous process was most prominent.

**1.1.2 Target Muscle Segmentation**

Images were automatically segmented using uAI software (United Imaging Intelligence, Shanghai, China). Trained image readers reviewed the automated segmentation results case by case, with manual correction when necessary. The target skeletal muscles at L3 comprised the abdominal wall muscles (transversus abdominis, internal and external obliques, and rectus abdominis), psoas muscles, and paraspinal muscles (erector spinae and quadratus lumborum).

**1.1.3 CT Attenuation Thresholding**

To distinguish skeletal muscle pixels from adjacent adipose tissue, including subcutaneous, visceral, and intermuscular fat, the CT attenuation range for skeletal muscle pixels was restricted to −29 to +150 Hounsfield units (HU), consistent with validated CT-based muscle assessment methods (1). Within the target anatomical region, pixels below −29 HU, generally representing macroscopic adipose tissue, and those above +150 HU, generally representing bone, vascular calcification, or contrast material, were automatically excluded by the algorithm.

**1.1.4 Calculation of Core Exposure Metrics**

Based on the segmentation and attenuation thresholding described above, the system automatically generated the following parameters:

Skeletal muscle cross-sectional area (CSA): the total area of skeletal muscle pixels within the specified attenuation range, expressed in cm².

Skeletal muscle index (SMI): CSA divided by height squared (CSA/height²), expressed in cm²/m², providing a height-normalized measure of skeletal muscle quantity.

Skeletal muscle density (SMD): the mean CT attenuation of the segmented skeletal muscle area, expressed in Hounsfield units (HU). SMD was used as an imaging marker of muscle composition. Lower SMD is commonly associated with greater lipid infiltration within skeletal muscle or myosteatosis (2), but it does not directly quantify microscopic fat infiltration.

**1.1.5 Imaging Quality Control**

During initial screening, patients with severe metal artifacts at L3 that interfered with attenuation measurement were excluded according to prespecified criteria to reduce exposure-measurement error.

**1.2 CCI Calculation and Event-Recording Rules**

This section describes the CCI data-ascertainment window, Clavien–Dindo grading, CCI calculation, and complication-recording rules from completion of liver transplantation to immediately before the postoperative day 90 landmark.

**1.2.1 Observation Window and Basic Scoring Rules**

The complication-observation window began at completion of liver transplantation (postoperative day 0 [POD 0]) and ended immediately before the 90-day landmark. Readmissions or emergency-department events occurring after discharge and before the postoperative day 90 landmark were included in the complication review when they met the prespecified definitions of postoperative complications.

All complications were graded according to the Clavien–Dindo (CD) classification (3). The CCI uses a nonlinear algorithm to integrate all complications and their CD grades into a continuous score from 0 to 100 (4); the primary analysis used the official CCI calculator (www.assessurgery.com).

The primary analysis included all CD grade I or higher complications that met prespecified diagnostic criteria and were supported by explicit medical-record documentation. A single CD grade I event corresponds to a CCI value of 8.7.

**1.2.2 Independence of Complication Events and Escalation-Recording Rules**

Distinct complications that separately met prespecified diagnostic criteria and could be independently identified were entered separately according to standard CCI rules; they were not merged solely because they might be pathophysiologically related.

When treatment of the same clinical complication escalated during a single episode, only the final highest CD grade reached by that event was recorded.

**1.2.3 Blinded Clinical Review and Adjudication**

Two attending physicians, each blinded to the patient's preoperative SMI and SMD measurements and post-landmark survival outcome, independently reviewed the complete medical record from completion of liver transplantation to immediately before the postoperative day 90 landmark and made initial determinations of complication diagnoses and CD grades. When their initial determinations differed, a third associate chief physician reviewed the source records and adjudicated the disagreement to establish the final complication record. The adjudicated CCI record was then compared with the primary analysis database on a patient-by-patient basis, and validation was summarized as the exact agreement rate. This rate describes agreement between the final adjudicated CCI verification record and the primary analysis database; it is not a measure of inter-rater reliability between the two initial reviewers before adjudication.

**Section 2: Causal Framework and Identifiability Assumptions**

**2.1 Conceptual Causal Framework and Prespecified Covariate Specification**

A directed acyclic graph (DAG) was developed using causal-diagram theory (5) to represent the temporal ordering of variables and the prespecified causal assumptions without imposing a parametric model on those relationships.

**2.1.1 DAG Topology and Temporal Ordering**

The expanded conceptual DAG was organized according to the temporal sequence of the study variables: preoperative SMI or SMD as the exposure, cumulative CCI within the prespecified mediator-observation window as the mediator, and all-cause mortality during the prespecified 5-year analysis window after the 90-day postoperative landmark as the outcome (Figure 2). SMI and SMD were analyzed in separate models; the combined exposure node in the diagram denotes the alternative exposure used in each model, not simultaneous inclusion of both exposures in one model.

Hepatocellular carcinoma (HCC) status, Eastern Cooperative Oncology Group (ECOG) performance status, and Model for End-Stage Liver Disease (MELD) 3.0 score are shown as the prespecified primary measured covariates. Age, sex, pre-existing non-hepatic comorbidity and metabolic burden, and donor graft characteristics were included as additional conceptual factors to represent potential causal pathways and sources of residual confounding. The pre-existing non-hepatic comorbidity and metabolic burden node represents a conceptual clinical domain rather than a single composite score constructed and entered into the primary models.

Arrows denote causal directions prespecified on the basis of temporal ordering, clinical knowledge, and prior literature. The DAG was used to clarify the study assumptions and potential identification limitations. It does not imply that every factor shown was included in the primary models, nor does it establish that HCC status, ECOG performance status, and MELD 3.0 score block all potential backdoor paths.

**2.1.2 Prespecified Primary Measured Covariates and Model Parsimony**

The 90-day landmark cohort had 56 deaths during the prespecified 5-year analysis window; including too many parameters in the Cox outcome model could therefore increase estimation instability and overfitting. On the basis of the prespecified conceptual causal framework, clinical relevance, data availability, and model parsimony, HCC status, ECOG performance status, and MELD 3.0 score were specified before fitting the primary mediation models as the measured covariates and were included in both the mediator and outcome models. HCC status represented baseline oncologic status, ECOG performance status represented baseline functional status, and MELD 3.0 represented the physiological severity of end-stage liver disease (6).

Each primary Cox outcome model contained one exposure, CCI, and the three measured covariates, for a total of five regression coefficients. With 56 outcome events, the descriptive events-per-variable (EPV) was 11.2. EPV was used only to describe model complexity and potential overfitting risk; it does not demonstrate correct model specification, sufficient identification of causal effects, or the ability of the covariate set to block all confounding paths (7). The covariate set was not assumed to be a minimally sufficient adjustment set under the expanded conceptual DAG, and residual or unmeasured confounding could not be excluded.

**2.1.3 Additional Conceptual Factors and Residual Confounding**

The expanded conceptual DAG also included age, sex, pre-existing non-hepatic comorbidity and metabolic burden, and donor graft characteristics. These nodes represent additional factors that may contribute to exposure-mediator, exposure-outcome, or mediator-outcome relationships; they do not indicate that all such factors were entered simultaneously into the primary models.

Age was examined to a limited extent in a prespecified covariate-substitution analysis that replaced ECOG performance status with age. Sex was examined exploratorily by testing exposure-by-sex interaction terms separately in the mediator and outcome models. Pre-existing non-hepatic comorbidity and metabolic burden, nutritional status, systemic inflammation, donor quality, surgical technique, and other perioperative factors were not comprehensively included in the primary models; related residual or unmeasured confounding therefore could not be excluded.

Unmeasured confounding was not represented as a single latent node in Figure 2. Its potential influence is discussed in Supplementary Table S5 separately for exposure-outcome, exposure-mediator, mediator-outcome, and exposure-induced mediator-outcome confounding. The mediational E-value was used only for exploratory assessment of unmeasured mediator-outcome confounding and does not evaluate all unmeasured confounding pathways.

**2.1.4 Clinical and Literature Basis for the Conceptual DAG**

The directed edges in Figure 2 were not generated from statistical significance within this cohort or from data-driven variable selection. They were prespecified according to temporal ordering, clinical plausibility, and prior literature. The diagram is a simplified conceptual causal framework and is not intended to exhaust all possible direct or indirect causal relationships.

Age and sex were included in the conceptual DAG as preoperative baseline factors. Age may be related to pre-existing non-hepatic comorbidity and metabolic burden, skeletal muscle phenotype, and long-term mortality risk. Sex may affect skeletal muscle phenotype and may contribute to the representation of liver-disease severity through the sex-related component of MELD 3.0. These nodes were included to clarify potential baseline structure, not to imply that their effects were fully controlled in the primary models.

Nutritional, metabolic, and inflammatory abnormalities associated with end-stage liver disease, including hyperammonemia, may promote skeletal muscle protein breakdown and inhibit protein synthesis (8–10). HCC-related tumor burden and wasting may be associated with lower skeletal muscle reserve and may affect long-term survival (11). Metabolic syndrome, insulin resistance, and fatty liver disease may also be related to intramuscular lipid accumulation and impaired muscle function (12, 13). Accordingly, HCC status, MELD 3.0 score, and pre-existing non-hepatic comorbidity and metabolic burden were represented in the conceptual diagram as baseline factors that may affect selected study variables.

Donor graft characteristics and incompletely measured surgical, perioperative, and early postoperative factors may affect both postoperative complication burden and long-term survival and therefore constitute potential sources of mediator-outcome confounding (14, 15). CCI was used to jointly quantify the number and severity of postoperative complications within the mediator-observation window. Previous studies have associated higher CCI or greater postoperative complication burden with poorer graft and patient outcomes after liver transplantation (16, 17). In this study, CCI was specified as the observed mediator, not as a surrogate endpoint for another unobserved biological mechanism.

ECOG performance status was used to represent baseline functional status and activity level, not as a formal frailty measure. Previous liver-transplantation studies have associated functional reserve and frailty with candidate and post-transplant outcomes (18). ECOG performance status was therefore prespecified for inclusion in the primary models and represented in the conceptual DAG as a baseline factor that may affect preoperative skeletal muscle phenotype, postoperative complication burden, and long-term survival.

**2.2 Identifiability Assumptions and Design Considerations**

Within the counterfactual causal-inference framework, identification and decomposition of natural direct effects (NDEs) and natural indirect effects (NIEs) from observational data require assumptions that cannot be fully verified from the observed data (19). The primary analysis included the prespecified measured covariates, but this covariate set was not assumed to block all confounding paths in the expanded conceptual DAG. The four core assumptions listed in Supplementary Table S5 are described below together with the principal concern, the design or analytical response, and the residual limitation in this study.

**Assumption 1: No unmeasured exposure-outcome confounding
Definition and concern:** Conditional on the measured covariates, there is no unmeasured common cause of both preoperative skeletal muscle phenotype and all-cause mortality during the prespecified 5-year post-landmark analysis window. Frailty, nutritional status, systemic inflammation, and pre-existing non-hepatic comorbidity and metabolic burden may affect both skeletal muscle phenotype and long-term survival.

**Design or analytical response:** The primary models included the prespecified HCC status, ECOG performance status, and MELD 3.0 score. The expanded conceptual DAG also represents age and pre-existing non-hepatic comorbidity and metabolic burden as potential sources of exposure-outcome confounding. Replacing ECOG performance status with age provided a limited assessment of sensitivity to an alternative measured-covariate specification.

**Residual limitation:** Residual and unmeasured exposure-outcome confounding could not be excluded. The age-substitution analysis did not include age and ECOG performance status simultaneously and therefore cannot verify this identifiability assumption.

**Assumption 2: No unmeasured mediator-outcome confounding, conditional on exposure and measured covariates**
**Definition and concern:** Conditional on preoperative skeletal muscle phenotype and the measured covariates, there is no unmeasured common cause of both CCI within the mediator-observation window and post-landmark mortality. Donor-related factors, surgical technique, and perioperative and early postoperative factors may affect both postoperative complication burden and subsequent survival.

**Design or analytical response:** The prespecified measured covariates were included in both the mediator and outcome models. The landmark design was intended to place mediator measurement before outcome follow-up, and the delayed 120-day landmark analysis introduced an additional 30-day interval between the mediator-observation window and outcome follow-up. Perioperative and donor characteristics are reported in Supplementary Table S1. The exploratory mediational E-value quantified the approximate strength of association that an unmeasured mediator-outcome confounder would need to have with both CCI and the outcome to move the NIE point estimate, or the confidence limit closest to the null, to the null.

**Residual limitation:** The landmark and delayed-landmark analyses address temporal ordering, not mediator-outcome confounding itself. Donor-related factors, surgical technique, and perioperative and postoperative determinants were not comprehensively measured. The mediational E-value evaluates only unmeasured mediator-outcome confounding, relies on treating the hazard ratio (HR) as an approximate risk ratio, and cannot verify this identifiability assumption. Blinded complication review and adjudication may reduce CCI measurement error but cannot eliminate mediator-outcome confounding.

**Assumption 3: No unmeasured exposure-mediator confounding**
**Definition and concern:** Conditional on the measured covariates, there is no unmeasured baseline common cause of both preoperative skeletal muscle phenotype and postoperative complication burden. Frailty, nutritional status, systemic inflammation, liver-disease severity, and other baseline factors may affect both.

**Design or analytical response:** The primary models included the prespecified HCC status, ECOG performance status, and MELD 3.0 score. The covariate-substitution analysis replacing ECOG performance status with age and the HCC subgroup analysis provided limited assessments of sensitivity to alternative measured baseline structures.

**Residual limitation:** Nutritional status, systemic inflammation, frailty, and other factors that may affect both skeletal muscle phenotype and postoperative complications were not comprehensively measured; residual exposure-mediator confounding may therefore remain. These sensitivity analyses cannot establish conditional exchangeability or verify the absence of unmeasured confounding.

**Assumption 4: No mediator-outcome confounder affected by the exposure**
**Definition and concern:** There is no exposure-induced mediator-outcome confounder that is affected by the preoperative skeletal muscle phenotype and also affects postoperative CCI and long-term survival.

**Design or analytical response:** Preoperative SMI and SMD were measured before liver transplantation, CCI was accumulated during the prespecified postoperative mediator-observation window, and outcome follow-up began at the landmark. Variables occurring after exposure measurement and before or during mediator measurement were not added indiscriminately to the primary models solely to improve model fit, because conditioning on an exposure-induced mediator-outcome confounder can bias natural direct and indirect effects.

**Residual limitation:** This assumption cannot be tested directly using the available observational data. If an exposure-induced mediator-outcome confounder exists, estimates of the NDE and NIE may be biased and the standard natural-effects decomposition may not be identifiable.

**Section 3: Advanced Statistical Modeling and Robustness Protocols**

**3.1 Effect Decomposition and Nonparametric Bootstrapping**

Counterfactual causal mediation analysis was implemented using a joint regression-modeling framework. Because the mediator, CCI, was continuous, the primary analysis used an ordinary least squares (OLS) model for the exposure-mediator relation. The outcome model used Cox proportional hazards regression for time to all-cause mortality, with the Efron method for tied event times. SMI and SMD were analyzed in separate models and standardized as z scores within the primary 90-day landmark cohort. For each exposure, the mediator model included CCI as the dependent variable and the standardized exposure, HCC status, ECOG performance status, and MELD 3.0 score as predictors. The Cox outcome model included the standardized exposure, CCI, and the same prespecified primary measured covariates. The primary models did not include an exposure-by-CCI interaction; this interaction was evaluated only in a prespecified sensitivity analysis. The exposure contrast was defined as a 1-standard deviation decrease.

Regression-based counterfactual mediation analysis was implemented using regmedint (20, 21) to estimate model-based NDE, NIE, and total-effect (TE) contrasts for a 1-standard deviation decrease in preoperative SMI or SMD in relation to all-cause mortality during the prespecified 5-year analysis window after the 90-day postoperative landmark.

Because CCI was right-skewed and the mediation effects were nonlinear functions derived from parameters in multiple models, formal uncertainty estimation used patient-level nonparametric bootstrapping. The mediator and outcome models were refitted and the NDE, NIE, and TE were recalculated in each resample. Percentile-based 95% confidence intervals (CIs) were constructed from 1,000 bootstrap resamples. Intervals based on asymptotic standard errors were used only for model checking.

The conventional proportion mediated (PM) is a ratio measure and can yield highly unstable estimates when the TE is close to the null or changes direction across bootstrap resamples. PM was therefore not used as a formally reported measure; the primary results were limited to the NDE, NIE, and TE.

**3.2 Robustness and Sensitivity Analyses**

The following prespecified robustness and sensitivity analyses were conducted to evaluate the sensitivity of the results to covariate specification, exposure-mediator interaction, CCI modeling, analysis population, and landmark specification:

1. ECOG performance status was replaced with age; both the mediator and outcome models included HCC status, age, and MELD 3.0 score.
2. An exposure-by-CCI interaction was added to the Cox outcome model, and nested models with and without the interaction were compared using a likelihood-ratio test.
3. CCI was dichotomized at the sample 75th percentile: CCI > 52.7 was defined as high CCI and CCI ≤ 52.7 as non-high CCI. A logistic regression model was used for the binary mediator.
4. Within the HCC subgroup, SMI and SMD were re-standardized and both the mediator and outcome models included ECOG performance status and MELD 3.0 score. HCC status was omitted because every patient in the subgroup had HCC.
5. Effect modification by sex was assessed exploratorily. In the mediator model, nested models with and without an exposure-by-sex interaction were compared using an F test; a likelihood-ratio test was used in the Cox outcome model. The full models retained the main effects of both exposure and sex (22).
6. The start of outcome follow-up was delayed to postoperative day 120. CCI accumulated from completion of liver transplantation to immediately before the postoperative day 90 landmark remained the mediator, and the SMI and SMD standardization scales from the primary 90-day landmark cohort were retained.
7. Because the dataset did not contain a mutually exclusive variable distinguishing specific underlying liver-disease etiologies, HCC status was evaluated only as an available broad disease/indication category. For each exposure, the reduced linear mediator model included the full-cohort standardized exposure, HCC status, ECOG performance status, and MELD 3.0 score. The corresponding interaction model additionally included the exposure-by-HCC-status term. Reduced and interaction models were compared using nested-model F tests. Adjusted HCC-status-specific slopes were derived from the interaction models and expressed as the mean difference in 90-day CCI associated with a 1-standard deviation decrease in SMI or SMD.

**3.3 Model Diagnostics**
The proportional hazards assumption for the Cox models was assessed using scaled Schoenfeld residuals, variable-specific tests, and global tests (23). OLS mediator models were evaluated graphically using studentized residual-versus-fitted plots and normal Q–Q plots, and heteroscedasticity was assessed with the Breusch–Pagan test. The Shapiro–Wilk test assessed normality using raw model residuals; studentized residuals, leverage values, and Cook's distance were also examined. In diagnostic testing, P > 0.05 indicates only that no statistical evidence of the corresponding assumption violation was detected; it does not establish that the assumption holds.

**3.4 Sensitivity to Unmeasured Mediator-Outcome Confounding: Mediational E-values**
Exploratory mediational E-values were calculated only for the NIE (24, 25) to provide an approximate assessment of the potential impact of unmeasured mediator-outcome confounding. The analysis posited an unmeasured confounder associated with both CCI and post-landmark mortality conditional on the exposure and measured covariates, and quantified the strength of association that the confounder would need to have with the mediator and outcome, respectively, to move the NIE point estimate or its confidence limit closest to the null value of 1 to the null.

Because the E-value formula is defined on the risk-ratio scale, the NIE HR was treated as an approximate risk ratio. Effects greater than or equal to 1 were entered directly into the standard formula; effects below 1 were inverted before calculation. E-values were calculated for the NIE point estimate and for the limit of its nonparametric bootstrap 95% CI closest to 1. If the 95% CI crossed 1, the E-value for the confidence limit was defined as 1.

The mediational E-value assesses only unmeasured mediator-outcome confounding. It does not assess unmeasured exposure-outcome confounding, exposure-mediator confounding, or exposure-induced mediator-outcome confounding, and it cannot verify the other identifiability assumptions. Because mortality during the prespecified 5-year analysis window after the landmark was not rare and the HR was treated as an approximate risk ratio, the E-value results were interpreted as exploratory.

**3.5 Interpretation of Cox-Based Mediation Effects**

Closed-form mediation-effect decomposition with a Cox outcome model relies on a rare-outcome approximation. The NDE, NIE, and TE were therefore interpreted as approximate mediation effects on the HR scale, not as exact causal effects on the risk-ratio scale. Strict causal interpretation also depends on the identifiability assumptions listed in Supplementary Table S5 being satisfied. Because residual or unmeasured confounding could not be excluded, the findings should be interpreted as HR-scale approximate mediation effects conditional on the prespecified models and identifiability assumptions. The time-to-event analyses also rely on censoring assumptions; because 82 recipients were right-censored before 5 years and detailed reasons for censoring were not fully classifiable from the analysis dataset, informative censoring cannot be completely excluded, although much of the shorter follow-up reflected the available calendar time for more recent transplantations.

**3.6 External Sex-Specific Low-SMI Threshold Sensitivity Analysis**

To assess sensitivity of the SMI interpretation to an external sex-specific threshold, low SMI was defined using the FLEXIT/Carey cutoffs developed in adults with end-stage liver disease awaiting liver transplantation: <50 cm²/m² in men and <39 cm²/m² in women (26). These cutoffs were treated as an imaging-based low-SMI classification rather than a complete diagnosis of sarcopenia because muscle strength and physical performance were unavailable. No corresponding external-threshold analysis was performed for SMD because a universally accepted sex-specific SMD cutoff for this liver-transplant setting was unavailable.

Classification counts, deaths within the 5-year post-landmark analysis window, and 90-day CCI were summarized by sex. Survival was compared using Kaplan-Meier estimates, the log-rank test, and an unadjusted Cox model. The adjusted Cox model included low-SMI classification, sex, HCC status, ECOG performance status, and MELD 3.0 score. CCI was deliberately omitted from that prognostic model because it was the hypothesized intermediate variable. A corresponding adjusted linear model evaluated the mean difference in CCI associated with low-SMI classification.

**Supplementary Tables**

**Supplementary Table S1. Perioperative and donor characteristics according to 90-day landmark eligibility**

| **Perioperative or donor characteristic** | **Excluded before the 90-day landmark (n = 28)** | **Included in the 90-day landmark cohort (n = 205)** | **P value** |
| --- | --- | --- | --- |
| Cold ischemia time, h | 8.0 [7.0, 8.0] | 7.0 [6.0, 8.0] | 0.194 |
| Estimated blood loss, mL | 2000.0 [1500.0, 3000.0] | 1500.0 [1000.0, 2500.0] | 0.016 |
| Anhepatic phase, min | 61.5 [54.0, 82.0] | 63.0 [54.0, 72.0] | 0.781 |
| Portal vein clamping time, min | 15.0 [10.0, 21.0] | 15.0 [10.0, 20.0] | 0.758 |
| Inferior vena cava clamping time, min | 50.5 [44.0, 56.0] | 49.0 [44.0, 55.0] | 0.473 |
| Donor age, years | 33.7 (14.8) | 38.6 (12.4) | 0.060 |

Data are presented as median [IQR] except donor age, which is presented as mean (SD). IQR, interquartile range.

P values were calculated using the Wilcoxon rank-sum test for cold ischemia time, estimated blood loss, anhepatic phase, portal vein clamping time, and inferior vena cava clamping time, and Welch’s t test for donor age.

Recipients were grouped according to eligibility for the postoperative day-90 landmark. The group excluded before the landmark included recipients who died before day 90 or whose recorded follow-up ended before day 90.

**Supplementary Table S2. Overview of robustness and sensitivity analyses**

| **Analysis** | **Methodological modification or diagnostic** | **SMI result** | **SMD result** | **Interpretation** |
| --- | --- | --- | --- | --- |
| Primary analysis | 90-day landmark; prespecified 5-year analysis window; adjusted for HCC, ECOG, and MELD 3.0 (n = 205; deaths = 56) | 1.155 (1.033–1.326) | 1.190 (1.062–1.382) | Reference estimates for comparison. |
| Covariate substitution | Age replaced ECOG among the prespecified measured covariates (n = 205; deaths = 56) | 1.160 (1.037–1.324) | 1.189 (1.064–1.382) | NIE estimates were similar after replacing ECOG with age. |
| Proportional-hazards assessment | Global Schoenfeld-residual test for each Cox outcome model | Global P = 0.544 | Global P = 0.603 | No statistical evidence of violation of the proportional-hazards assumption in either model. |
| Exposure×CCI interaction in the outcome model | Likelihood-ratio test comparing Cox models with and without exposure×CCI | LRT P = 0.823 | LRT P = 0.155 | No statistical evidence of an exposure×CCI interaction in either outcome model. |
| Mediator recoding | CCI dichotomized at the 75th percentile: >52.7 (high n = 51; remaining n = 154) | 1.114 (0.992–1.379) | 1.140 (1.020–1.373) | Dichotomization attenuated the estimates and widened uncertainty; the SMI interval included 1, whereas the SMD interval remained above 1. |
| HCC subgroup | HCC-only subgroup; SMI and SMD re-standardized within subgroup (n = 113; deaths = 39) | 1.089 (0.952–1.320) | 1.148 (0.993–1.400) | Directionally similar estimates with wider subgroup intervals; both intervals included 1. |
| Sex effect modification | Exploratory exposure×sex tests in the Cox outcome model and linear mediator model | Mediator P = 0.746; outcome P = 0.342 | Mediator P = 0.445; outcome P = 0.295 | No statistical evidence of effect modification by sex in either the outcome or mediator pathway. |
| Delayed landmark | Outcome follow-up began at postoperative day 120, using CCI measured through day 90 (n = 200; deaths = 54) | 1.155 (1.035–1.317) | 1.187 (1.056–1.364) | NIE estimates remained similar after introducing a 30-day buffer between mediator ascertainment and outcome follow-up. |

For rows reporting mediation effects, results are HR-scale approximate natural indirect effects for a 1-SD decrease in SMI or SMD, with percentile-bootstrap 95% confidence intervals based on 1,000 nonparametric resamples.

The proportional-hazards and exposure×CCI rows report diagnostic P values rather than mediation-effect estimates. P > 0.05 indicates that no statistically significant departure was detected; it does not prove that the corresponding assumption is true.

For mediator recoding, CCI_high was defined as 1 only when CCI exceeded the sample 75th percentile (52.7).

In the HCC subgroup, SMI and SMD were standardized within the subgroup; the reported contrast therefore represents a 1-SD decrease within that subgroup.

The sex-interaction analysis was exploratory. Outcome-pathway P values were obtained by likelihood-ratio tests, and mediator-pathway P values were obtained by nested-model F tests.

The 120-day delayed-landmark analysis used the CCI accumulated through postoperative day 90 and began outcome follow-up at day 120; it was not an analysis of a 120-day cumulative CCI.

CCI, Comprehensive Complication Index; CI, confidence interval; ECOG, Eastern Cooperative Oncology Group; HCC, hepatocellular carcinoma; LRT, likelihood-ratio test; MELD, Model for End-Stage Liver Disease; NIE, natural indirect effect; SMD, skeletal muscle density; SMI, skeletal muscle index.

**Supplementary Table S3. HCC subgroup mediation analysis and exploratory effect modification by HCC status**

**Panel A. Causal mediation analysis in the HCC subgroup (n = 113)**

| **Exposure contrast** | **Effect** | **Measure** | **Estimate (95% bootstrap CI)** |
| --- | --- | --- | --- |
| Skeletal muscle index (SMI), 1-SD decrease | Natural indirect effect (NIE) | Hazard ratio | 1.089 (0.952–1.320) |
|  | Natural direct effect (NDE) | Hazard ratio | 1.335 (0.918–2.129) |
|  | Total effect (TE) | Hazard ratio | 1.453 (1.047–2.252) |
| Skeletal muscle density (SMD), 1-SD decrease | Natural indirect effect (NIE) | Hazard ratio | 1.148 (0.993–1.400) |
|  | Natural direct effect (NDE) | Hazard ratio | 1.083 (0.694–1.736) |
|  | Total effect (TE) | Hazard ratio | 1.243 (0.817–2.029) |

NIE, NDE, and TE are reported as hazard ratios for all-cause mortality during the prespecified 5-year analysis window after the 90-day postoperative landmark.

Values in parentheses are percentile-bootstrap 95% confidence intervals based on 1000 nonparametric bootstrap resamples.

The 90-day Comprehensive Complication Index was specified as the mediator. Models were adjusted for ECOG performance status and MELD 3.0 score; HCC was not included as a covariate because all patients in this subgroup had HCC.

SMI and SMD were re-standardized within the HCC subgroup, so each contrast represents a 1-SD decrease within this subgroup.

CCI, Comprehensive Complication Index; CI, confidence interval; ECOG, Eastern Cooperative Oncology Group; HCC, hepatocellular carcinoma; MELD, Model for End-Stage Liver Disease; NDE, natural direct effect; NIE, natural indirect effect; SMD, skeletal muscle density; SMI, skeletal muscle index; TE, total effect.

**Panel B. Exploratory effect modification of the association with 90-day CCI by HCC status (n = 205)**

| **Exposure** | **HCC status** | **n** | **Adjusted mean difference in 90-day CCI per 1-SD decrease (95% CI)** | **P value** | **P for interaction** |
| --- | --- | --- | --- | --- | --- |
| SMI | Non-HCC | 92 | 4.048 (1.023–7.073) | 0.009 | 0.408 |
|  | HCC | 113 | 5.700 (3.171–8.228) | <0.001 |  |
| SMD | Non-HCC | 92 | 3.876 (0.909–6.843) | 0.011 | 0.218 |
|  | HCC | 113 | 6.304 (3.819–8.790) | <0.001 |  |

SMI and SMD were standardized within the full postoperative day-90 landmark cohort. Models included the standardized exposure, HCC status, ECOG performance status, MELD 3.0 score, and the corresponding exposure-by-HCC-status interaction term. Positive estimates indicate a higher adjusted 90-day CCI associated with a 1-SD decrease in SMI or SMD. Interaction P values were obtained from nested-model F tests.

HCC status represents an available broad disease/indication category and not a complete classification of underlying liver-disease etiology. CCI, Comprehensive Complication Index; CI, confidence interval; ECOG, Eastern Cooperative Oncology Group; HCC, hepatocellular carcinoma; MELD, Model for End-Stage Liver Disease; SMD, skeletal muscle density; SMI, skeletal muscle index.

**Supplementary Table S4. Sensitivity analysis using a delayed 120-day outcome landmark**

| **Analysis** | **Outcome follow-up start** | **Analysis cohort, n** | **Deaths within 5 years, n** | **SMI NIE, HR (95% bootstrap CI)** | **SMD NIE, HR (95% bootstrap CI)** |
| --- | --- | --- | --- | --- | --- |
| **Primary 90-day landmark** | Postoperative day 90 | 205 | 56 | 1.155 (1.033–1.326) | 1.190 (1.062–1.382) |
| Delayed 120-day landmark | Postoperative day 120 | 200 | 54 | 1.155 (1.035–1.317) | 1.187 (1.056–1.364) |

Both analyses used the Comprehensive Complication Index accumulated through postoperative day 90 as the mediator. Outcome follow-up began at day 90 in the primary analysis and at day 120 in the delayed-landmark analysis.

Results are natural indirect-effect hazard ratios for a 1-SD decrease in SMI or SMD, with percentile-bootstrap 95% confidence intervals based on 1000 nonparametric bootstrap resamples.

All models were adjusted for hepatocellular carcinoma, ECOG performance status, and MELD 3.0 score. The 120-day analysis retained the SMI and SMD standardization scales from the primary 90-day cohort, so the exposure contrasts were directly comparable.

A 60-day landmark analysis was not performed because the 90-day mediator had not been completely ascertained by postoperative day 60; beginning outcome follow-up at day 60 would violate the temporal ordering required for mediation analysis.

CCI, Comprehensive Complication Index; CI, confidence interval; ECOG, Eastern Cooperative Oncology Group; MELD, Model for End-Stage Liver Disease; NIE, natural indirect effect; SMD, skeletal muscle density; SMI, skeletal muscle index.

**Supplementary Table S5. Identifiability assumptions for the causal mediation analysis**

| **Identifiability assumption** | **Study-specific concern** | **Design or analysis response** | **Residual limitation** |
| --- | --- | --- | --- |
| **1. No unmeasured exposure–outcome confounding** | Frailty, nutritional status, systemic inflammation, comorbidity, and other determinants of preoperative muscle phenotype may also influence long-term survival. | The primary models included the prespecified measured covariates HCC status, ECOG performance status, and MELD 3.0 score. The expanded conceptual DAG additionally represented age and pre-existing non-hepatic comorbidity and metabolic burden as potential sources of residual exposure–outcome confounding. Substitution of age for ECOG provided a limited assessment of sensitivity to an alternative measured covariate specification. | Residual and unmeasured exposure–outcome confounding cannot be excluded. The age-substitution analysis did not jointly include age and ECOG and therefore cannot validate this assumption. |
| **2. No unmeasured mediator–outcome confounding, conditional on exposure and measured covariates** | Donor, technical, perioperative, or early postoperative factors may affect both postoperative complication burden during the mediator ascertainment window and subsequent survival. | Prespecified measured covariates were included in both the mediator and outcome models. The landmark design was intended to order mediator ascertainment before outcome follow-up, and the 120-day delayed-landmark analysis introduced an additional 30-day buffer. Perioperative and donor characteristics were described in Supplementary Table S1. Exploratory mediational E-values quantified the approximate strength of associations that an unmeasured mediator–outcome confounder would need to have with the mediator and outcome to move the NIE point estimate, or the confidence-interval limit closest to the null, to the null. | Landmarking and the delayed-landmark analysis address temporal ordering rather than mediator–outcome confounding. Donor, technical, perioperative, and postoperative determinants were not comprehensively measured. Mediational E-values were exploratory, relied on treating hazard ratios as approximate risk ratios, addressed only unmeasured mediator–outcome confounding, and did not validate the identifying assumption. |
| **3. No unmeasured exposure–mediator confounding** | Frailty, nutritional status, systemic inflammation, liver-disease severity, and other baseline factors may influence both preoperative muscle phenotype and postoperative complication burden. | The primary models included the prespecified measured covariates HCC status, ECOG performance status, and MELD 3.0 score. The age-for-ECOG substitution analysis and the HCC subgroup analysis provided limited assessments of sensitivity to alternative measured baseline structures. | Frailty, nutritional status, systemic inflammation, and other determinants of muscle phenotype and postoperative complications were not comprehensively measured. Residual exposure–mediator confounding therefore remains possible, and the sensitivity analyses do not establish conditional exchangeability. |
| **4. No mediator–outcome confounder affected by the exposure** | Post-exposure variables influenced by muscle phenotype could subsequently affect both postoperative complication burden and survival, creating exposure-induced mediator–outcome confounding. | Variables potentially occurring after the exposure and before or during mediator ascertainment were not included merely to improve model fit, because conditioning on exposure-induced mediator–outcome confounders can bias natural direct and indirect effects. The prespecified temporal sequence was preoperative muscle phenotype, postoperative complication burden during the mediator ascertainment window, and survival after the landmark. | This assumption is not empirically testable with the available data. If an exposure-induced mediator–outcome confounder exists, the natural direct and indirect effect estimates may be biased, and the standard natural-effect decomposition may not be identified. |

These assumptions are required for a causal interpretation of the natural direct and indirect effects and cannot be verified from the observed data alone.

The listed design features, model specifications, subgroup analyses, delayed-landmark analysis, and mediational E-values should be interpreted as design or sensitivity procedures rather than evidence that an identification assumption holds.

Mediational E-values address only unmeasured mediator–outcome confounding. Because mortality during the prespecified 5-year analysis window after the landmark was not rare, hazard ratios were treated as approximate risk ratios, and the E-value results should be interpreted as exploratory.

CCI, Comprehensive Complication Index; DAG, directed acyclic graph; ECOG, Eastern Cooperative Oncology Group; HCC, hepatocellular carcinoma; MELD, Model for End-Stage Liver Disease; NDE, natural direct effect; NIE, natural indirect effect.

**Supplementary Table S6. Sex-specific SMI/SMD distributions and exploratory tests for effect modification by sex (n = 205)**

**Panel A. Sex-specific absolute SMI and SMD distributions**

| **Sex** | **n** | **SMI (cm²/m²)** | | | **SMD (HU)** | | |
| --- | --- | --- | --- | --- | --- | --- | --- |
|  |  | **Mean ± SD** | **Median (IQR)** | **Minimum–maximum** | **Mean ± SD** | **Median (IQR)** | **Minimum–maximum** |
| Male | 177 | 50.12 ± 8.31 | 50.66 (43.97–55.31) | 30.17–76.51 | 39.14 ± 6.43 | 39.58 (33.99–43.18) | 24.87–54.60 |
| Female | 28 | 48.55 ± 7.71 | 48.30 (43.35–54.45) | 34.32–62.02 | 38.86 ± 5.83 | 39.14 (36.56–42.30) | 22.68–51.79 |

Values are mean ± SD, median (interquartile range), or minimum–maximum, as indicated. These summaries are descriptive; no between-sex hypothesis test was prespecified.

**Panel B. Exploratory tests for effect modification by sex**

| **Exposure metric** | **Evaluated pathway** | **Interaction term** | **Model-comparison test** | **Interpretation** |
| --- | --- | --- | --- | --- |
| Skeletal muscle index (SMI) | Mediator model: 90-day CCI | SMI × sex | Nested F test; P = 0.746 | No statistical evidence of interaction. |
|  | Outcome model: 5-year mortality after the 90-day landmark | SMI × sex | LRT; P = 0.342 | No statistical evidence of interaction. |
| Skeletal muscle density (SMD) | Mediator model: 90-day CCI | SMD × sex | Nested F test; P = 0.445 | No statistical evidence of interaction. |
|  | Outcome model: 5-year mortality after the 90-day landmark | SMD × sex | LRT; P = 0.295 | No statistical evidence of interaction. |

Sex interaction was evaluated separately in the linear mediator model for the 90-day Comprehensive Complication Index and in the Cox outcome model for all-cause mortality during the prespecified 5-year analysis window after the 90-day landmark.

Mediator-model P values were obtained from nested-model F tests. Outcome-model P values were obtained from likelihood-ratio tests comparing Cox models with and without the exposure×sex interaction term.

All models were adjusted for hepatocellular carcinoma, ECOG performance status, and MELD 3.0 score. The sex-interaction dataset included all 205 patients in the primary analysis cohort with nonmissing sex. These analyses were exploratory.

P > 0.05 indicates that no statistically significant interaction was detected; it does not establish absence of effect modification, and limited power to detect interaction should be considered.

CCI, Comprehensive Complication Index; ECOG, Eastern Cooperative Oncology Group; LRT, likelihood-ratio test; MELD, Model for End-Stage Liver Disease; SMD, skeletal muscle density; SMI, skeletal muscle index.

**Supplementary Table S7. External sex-specific low-SMI threshold sensitivity analysis**

**Panel A. Classification counts and 90-day CCI by sex**

| **Sex** | **SMI classification** | **n** | **Deaths within 5 years, n** | **90-day CCI, median (IQR)** |
| --- | --- | --- | --- | --- |
| Male | Not low SMI | 94 | 25 | 37.2 (29.6–47.4) |
| Male | Low SMI | 83 | 24 | 46.8 (37.2–59.7) |
| Female | Not low SMI | 25 | 7 | 48.1 (39.5–52.6) |
| Female | Low SMI | 3 | 0 | 55.8 (54.6–59.5) |

**Panel B. Associations with 90-day CCI and 5-year post-landmark mortality**

| **Analysis** | **Effect measure** | **Estimate (95% CI)** | **P value** |
| --- | --- | --- | --- |
| Kaplan-Meier log-rank test | P value only | — | 0.734 |
| Unadjusted Cox mortality model | Hazard ratio | 1.096 (0.646–1.861) | 0.734 |
| Adjusted Cox mortality model | Hazard ratio | 1.293 (0.727–2.298) | 0.382 |
| Adjusted linear CCI model | Mean CCI difference | 7.900 (3.731–12.068) | <0.001 |

**Panel C. Five-year Kaplan-Meier estimates**

| **SMI classification** | **Baseline n** | **Deaths within 5 years, n** | **At risk at 5 years, n** | **5-year survival (95% CI), %** |
| --- | --- | --- | --- | --- |
| Not low SMI | 119 | 32 | 39 | 64.4 (54.8–75.6) |
| Low SMI | 86 | 24 | 28 | 66.1 (55.6–78.6) |

Low SMI was defined as SMI <50 cm²/m² in men and <39 cm²/m² in women. The contrast in Panel B is low SMI versus not low SMI. The adjusted Cox and linear models included sex, HCC status, ECOG performance status, and MELD 3.0 score. Kaplan-Meier estimates in Panel C are survival estimates rather than crude proportions.

These external thresholds provide an imaging-based low-SMI sensitivity analysis and do not establish sarcopenia without muscle-strength and physical-performance assessment. No external SMD threshold was imposed. CCI, Comprehensive Complication Index; CI, confidence interval; ECOG, Eastern Cooperative Oncology Group; HCC, hepatocellular carcinoma; IQR, interquartile range; MELD, Model for End-Stage Liver Disease; SMI, skeletal muscle index.

**Supplementary Figure Legends**

**Supplementary Figure S1.** Exploratory mediational E-value sensitivity curves for the natural indirect effects (NIEs) of lower skeletal muscle index and skeletal muscle density through postoperative complication burden. Solid curves represent the NIE point estimates, and dashed curves represent the limits of the percentile-bootstrap 95% confidence intervals closest to the null. Circles and triangles indicate the corresponding mediational E-values. The horizontal and vertical axes represent the strengths of association that an unmeasured mediator–outcome confounder would need to have with the outcome and mediator, respectively, conditional on the exposure and measured covariates, to move the respective target estimate to the null. Because mortality during the prespecified 5-year analysis window was not rare, hazard ratios were treated as approximate risk ratios, and the results should be interpreted as exploratory. The analysis addresses sensitivity to unmeasured mediator–outcome confounding and does not assess all other identification assumptions.

**Supplementary Figure S2.** Assessment of the proportional-hazards assumption for the Cox outcome models within the prespecified 5-year analysis window after the 90-day landmark. Scaled Schoenfeld residual plots are shown for skeletal muscle index (SMI) and the Comprehensive Complication Index (CCI) in the SMI outcome model (Panels A and B), and for skeletal muscle density (SMD) and CCI in the SMD outcome model (Panels C and D). Gray points represent scaled Schoenfeld residuals, solid black curves represent smoothed time-dependent trends, dashed curves indicate approximate 95% confidence bands, and dotted horizontal lines denote zero. Variable-specific tests showed no statistically significant departures from proportional hazards for SMI, CCI in the SMI model, SMD, or CCI in the SMD model. Global tests also showed no evidence of violation for either the SMI outcome model (P = 0.544) or the SMD outcome model (P = 0.603). Both models were adjusted for hepatocellular carcinoma, Eastern Cooperative Oncology Group performance status, and Model for End-Stage Liver Disease 3.0 score.

**Supplementary Figure S3.** Residual diagnostics for the linear mediator models of the 90-day Comprehensive Complication Index. Panels A and B show the residuals-versus-fitted plot and normal Q–Q plot for the skeletal muscle index mediator model, and Panels C and D show the corresponding plots for the skeletal muscle density mediator model. Points in the residuals-versus-fitted plots represent studentized residuals, solid black curves represent LOESS-smoothed trends, the dotted horizontal lines denote zero, and the dashed horizontal lines denote studentized residuals of −2 and 2. The Q–Q plots are based on studentized residuals. Breusch–Pagan tests showed no evidence of heteroscedasticity in either model. The Shapiro–Wilk test, calculated using the raw model residuals, showed no evidence of non-normality for the skeletal muscle index model but indicated mild departure from normality for the skeletal muscle density model. Both models were adjusted for hepatocellular carcinoma, Eastern Cooperative Oncology Group performance status, and Model for End-Stage Liver Disease 3.0 score.

**Supplementary Figure S4.** Bootstrap distributions of the natural indirect effects for the skeletal muscle index (SMI) and skeletal muscle density (SMD) models. Histograms show estimates from 1,000 nonparametric bootstrap resamples. The dotted vertical line indicates the null value (HR = 1), the solid line indicates the point estimate, and the dashed lines indicate the percentile-bootstrap 95% confidence interval. Natural indirect effects (NIEs) are presented as HR-scale approximate mediation effects.

**Supplementary References**

1. Mitsiopoulos N, Baumgartner RN, Heymsfield SB, Lyons W, Gallagher D, Ross R. Cadaver validation of skeletal muscle measurement by magnetic resonance imaging and computerized tomography. J Appl Physiol (1985). (1998) 85:115–122. doi: 10.1152/jappl.1998.85.1.115.

2. Aubrey J, Esfandiari N, Baracos VE, Buteau FA, Frenette J, Putman CT, et al. Measurement of skeletal muscle radiation attenuation and basis of its biological variation. Acta Physiol (Oxf). (2014) 210:489–497. doi: 10.1111/apha.12224.

3. Dindo D, Demartines N, Clavien PA. Classification of surgical complications: a new proposal with evaluation in a cohort of 6336 patients and results of a survey. Ann Surg. (2004) 240:205–213. doi: 10.1097/01.SLA.0000133083.54934.AE.

4. Slankamenac K, Graf R, Barkun J, Puhan MA, Clavien PA. The Comprehensive Complication Index: a novel continuous scale to measure surgical morbidity. Ann Surg. (2013) 258:1–7. doi: 10.1097/SLA.0b013e318296c732.

5. Greenland S, Pearl J, Robins JM. Causal diagrams for epidemiologic research. Epidemiology. (1999) 10:37–48. doi: 10.1097/00001648-199901000-00008.

6. Kim WR, Mannalithara A, Heimbach JK, Kamath PS, Asrani SK, Biggins SW, et al. MELD 3.0: the Model for End-Stage Liver Disease updated for the modern era. Gastroenterology. (2021) 161:1887–1895.e4. doi: 10.1053/j.gastro.2021.08.050.

7. Vittinghoff E, McCulloch CE. Relaxing the rule of ten events per variable in logistic and Cox regression. Am J Epidemiol. (2007) 165:710–718. doi: 10.1093/aje/kwk052.

8. Dasarathy S, Merli M. Sarcopenia from mechanism to diagnosis and treatment in liver disease. J Hepatol. (2016) 65:1232–1244. doi: 10.1016/j.jhep.2016.07.040.

9. Ebadi M, Bhanji RA, Mazurak VC, Montano-Loza AJ. Sarcopenia in cirrhosis: from pathogenesis to interventions. J Gastroenterol. (2019) 54:845–859. doi: 10.1007/s00535-019-01605-6.

10. Davuluri G, Krokowski D, Guan BJ, Kumar A, Thapaliya S, Singh D, et al. Metabolic adaptation of skeletal muscle to hyperammonemia drives the beneficial effects of L-leucine in cirrhosis. J Hepatol. (2016) 65:929–937. doi: 10.1016/j.jhep.2016.06.004.

11. Meza-Junco J, Montano-Loza AJ, Baracos VE, Prado CMM, Bain VG, Beaumont C, et al. Sarcopenia as a prognostic index of nutritional status in concurrent cirrhosis and hepatocellular carcinoma. J Clin Gastroenterol. (2013) 47:861–870. doi: 10.1097/MCG.0b013e318293a825.

12. Watt KD, Charlton MR. Metabolic syndrome and liver transplantation: a review and guide to management. J Hepatol. (2010) 53:199–206. doi: 10.1016/j.jhep.2010.01.040.

13. Bhanji RA, Narayanan P, Allen AM, Malhi H, Watt KD. Sarcopenia in hiding: the risk and consequence of underestimating muscle dysfunction in nonalcoholic steatohepatitis. Hepatology. (2017) 66:2055–2065. doi: 10.1002/hep.29420.

14. Feng S, Goodrich NP, Bragg-Gresham JL, Dykstra DM, Punch JD, DebRoy MA, et al. Characteristics associated with liver graft failure: the concept of a donor risk index. Am J Transplant. (2006) 6:783–790. doi: 10.1111/j.1600-6143.2006.01242.x.

15. Kalisvaart M, de Haan JE, Polak WG, Metselaar HJ, Wijnhoven BPL, IJzermans JNM, et al. Comparison of postoperative outcomes between donation after circulatory death and donation after brain death liver transplantation using the Comprehensive Complication Index. Ann Surg. (2017) 266:772–778. doi: 10.1097/SLA.0000000000002419.

16. Lai Q, Melandro F, Nowak G, Nicolini D, Iesari S, Fasolo E, et al. The role of the Comprehensive Complication Index for the prediction of survival after liver transplantation. Updates Surg. (2021) 73:209–221. doi: 10.1007/s13304-020-00878-4.

17. Castanedo S, Toledo E, Fernández-Santiago R, Castillo F, Echeverri J, Rodríguez-Sanjuán JC. Influence of postoperative complications on long-term survival in liver transplant patients. World J Gastrointest Surg. (2020) 12:336–345. doi: 10.4240/wjgs.v12.i8.336.

18. Lai JC, Sonnenday CJ, Tapper EB, Duarte-Rojo A, Dunn MA, Bernal W, et al. Frailty in liver transplantation: an expert opinion statement from the American Society of Transplantation Liver and Intestinal Community of Practice. Am J Transplant. (2019) 19:1896–1906. doi: 10.1111/ajt.15392.

19. VanderWeele TJ. Mediation analysis: a practitioner’s guide. Annu Rev Public Health. (2016) 37:17–32. doi: 10.1146/annurev-publhealth-032315-021402.

20. VanderWeele TJ. Causal mediation analysis with survival data. Epidemiology. (2011) 22:582–585. doi: 10.1097/EDE.0b013e31821db37e.

21. Valeri L, VanderWeele TJ. Mediation analysis allowing for exposure-mediator interactions and causal interpretation: theoretical assumptions and implementation with SAS and SPSS macros. Psychol Methods. (2013) 18:137–150. doi: 10.1037/a0031034.

22. Benmassaoud A, Roccarina D, Arico FM, Cilla M, Donghia R, Leandro G, et al. Sex is a major effect modifier between body composition and mortality in patients with cirrhosis assessed for liver transplantation. Liver Int. (2023) 43:160–169. doi: 10.1111/liv.15293.

23. Grambsch PM, Therneau TM. Proportional hazards tests and diagnostics based on weighted residuals. Biometrika. (1994) 81:515–526. doi: 10.1093/biomet/81.3.515.

24. VanderWeele TJ, Ding P. Sensitivity analysis in observational research: introducing the E-value. Ann Intern Med. (2017) 167:268–274. doi: 10.7326/M16-2607.

25. Smith LH, VanderWeele TJ. Mediational E-values: approximate sensitivity analysis for unmeasured mediator-outcome confounding. Epidemiology. (2019) 30:835–837. doi: 10.1097/EDE.0000000000001064.

26. Carey EJ, Lai JC, Wang CW, Dasarathy S, Lobach I, Montano-Loza AJ, et al. A multicenter study to define sarcopenia in patients with end-stage liver disease. Liver Transpl. (2017) 23:625–633. doi: 10.1002/lt.24750.
